# Multi-omics comprehensive analyses of programmed cell death patterns to regulate the immune characteristics of head and neck squamous cell carcinoma

**DOI:** 10.1016/j.tranon.2023.101862

**Published:** 2024-01-18

**Authors:** Yi Jin, Siwei Huang, Hongyu Zhou, Zhanwang Wang, Yonghong Zhou

**Affiliations:** a Department of Radiation Oncology, Hunan Cancer Hospital, The Affiliated Cancer Hospital of Xiangya School of Medicine, Central South University, Changsha, Hunan 410013, China; b Key Laboratory of Translational Radiation Oncology, Department of Radiation Oncology, Hunan Cancer Hospital and The Affiliated Cancer Hospital of Xiangya School of Medicine, Central South University, Changsha 410013, China; c School of Humanities and Management, Hunan University of Chinese Medicine, Changsha, Hunan 410208, China; d Department of Oncology, Third Xiangya Hospital of Central South University, Changsha 410013, China; e School of Medicine, Shanghai University, 99 Shangda Road, Shanghai 200444, China

**Keywords:** Head and neck squamous cell carcinoma, Programmed cell death, Genetic heterogeneity, Machine learning, Immune-infiltrating characteristics

## Abstract

Head and neck squamous cell carcinoma (HNSCC) is a heterogeneous cancer with high morbidity and mortality. Triggering the programmed cell death (PCD) to enhance the anti-tumor therapies is being applied in multiple cancers. However, the limited understanding of genetic heterogeneity in HNSCC severely hampers the clinical efficacy. We systematically analyzed 14 types of PCD in HNSCC from The Cancer Genome Atlas (TCGA). We utilized ssGSEA to calculate the PCD scores and classify patients into two clusters. Subsequently, we displayed the genomic alteration landscape to unravel the significant differences in copy number alterations and gene mutations. Furthermore, we calculated the IC50 values of targeted drugs to predict the differences in sensitivity. To identify the immune-related prognostic types, we comprehensively estimated the relationship between immune indicators and all prognostic PCD in three datasets (TCGA, GSE65858, GSE41613). Finally, 7 regulators were filtered. Subsequently, we integrated 10 machine learning algorithms and 101 algorithm combinations to test the clinical predictive efficacy. Using WGCNA as a basis, we built a weighted co-expression network to identify modules involved in the immune landscape with different colors. Meanwhile, our results indicated that blue and red modules containing crucial regulators closely related to the CD4+, CD8+ *T* cells, TMB or PD-L1. FCGR2A from blue module, CSF2, INHBA, and THBS1 from the red module were determined. After verifying *in vivo* experiments, FCGR2A was identified as hub gene. In conclusion, our findings suggest a potential role of PCD in HNSCC, offering new insights into effective immunotherapy and anti-tumor therapies in HNSCC.

## Highlights

• This is a thorough comprehension of specific types of cell death in HNSCC.
• Our findings offer new insights into effective immunotherapy and anti-tumor therapies in HNSCC.
• FCGR2A is identified as hub gene to regulation of heterogeneity in HNSCC.

## Graphical abstract

**Figure:**
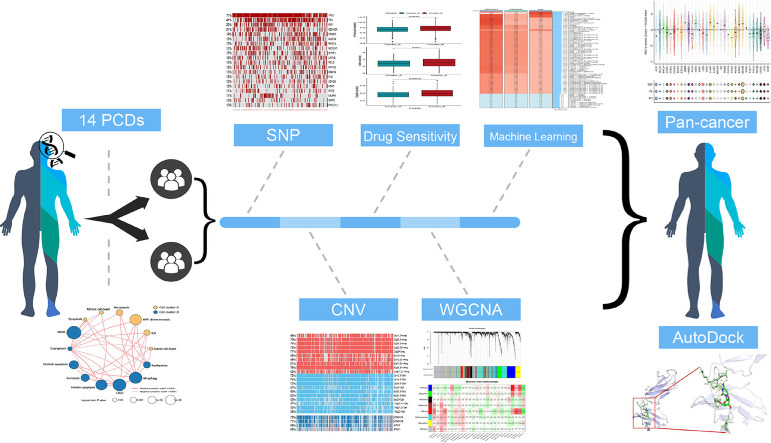

## Introduction

Head and neck squamous cell carcinoma (HNSCC) arises from the mucosal epithelium in the oral cavity, pharynx and larynx and is a heterogeneity pathological malignant cancer with the leading causes of morbidity and mortality worldwide [1]. It is reported that there were approximately 900,000 new cases in 2020 [2]. For patients in early stages, the main treatments of HNSCC are surgery, radiotherapy combined with chemotherapy, which may reach a high expectation of tumor control [3]. Regrettably, a delayed onset of a specific symptom may complicate diagnosis and result in an advanced stage with a lower survival rate. Epidermal growth factor receptor (EGFR) inhibitors and immune checkpoint inhibitors have emerged as novel and potent anti-tumor therapies in recent years. Moreover, several randomized phase 3 trials have confirmed that these inhibitors, when used in combination with chemotherapy, are an appropriate initial treatment for recurrent or metastatic disease [4,5]. However, because most patients do not respond well to immunotherapies and EGFR inhibitors, the prognostic significance of PD-L1/EGFR expression remains debatable. Because of the resistance and unfavorable prognosis associated with HNSCC, selecting an appropriate treatment remains a significant challenge and is often described as a “huge barrier”. Determining the precise molecular characterization and immunological profile of HNSCC is extremely important. This implies that integrating prognostic and predictive biomarkers into clinical care could promote longer lifespan by eliminating obstacles to targeted treatments [6].

Cell death is an important process in the growth and development, disease progression, and homeostasis of multicellular organisms [7]. In 2015, the Nomenclature Committee on Cell Death (NCCD) classified cell death into programmed cell death (PCD) [8]. With a focus on mechanistic and essential components, NCCD presented an enhanced taxonomy of cell death subroutines in 2018 [9]. Multiple types of cell death have been redefined, including intrinsic apoptosis, extrinsic apoptosis, mitochondrial permeability transition (MPT)-driven necrosis, necroptosis, ferroptosis, pyroptosis, parthanatos, entotic cell death, NETotic cell death (known as NETosis), lysosome-dependent cell death, autophagy-dependent cell death, and immunogenic cell death. For decades, these types of cell death have been shown to play a crucial role in the development and metastasis of malignant tumors and may explain a significant portion of clinical heterogeneity [10]. Currently, many cancer therapies aimed to trigger specific cell death are being developed and utilized in the treatment of various types of cancer. For example, HPV-positive HNSCC may be more sensitive to mitochondria-targeted treatments, such as mitocans. In addition, the mesenchymal (MS) subgroup of HNSCC, which has an elevated expression of EMT-associated genes, could be the most sensitive to ferroptosis. However, the presence of genetic heterogeneity and the influence of the tumor microenvironment (TME) severely limit the clinical efficacy of these approaches [10].

Therefore, a thorough comprehension of the sensitivity or resistance to specific types of cell death, as influenced by the specific genetic microenvironment that occurs during the pathogenesis of HNSCC, may uncover targets for innovative therapeutic strategies. In summary, our study uncovers the clinical heterogeneity among HNSCC patients and reveals the landscape of cell death, which could assist in selecting appropriate therapeutic regimens for HNSCC patients.

## Materials and methods

### Data download and collection

RNA sequencing (raw transcriptome counts data) and clinical information, including age, sex, and TNM stage, were downloaded from the publicly available data sources, such as the TCGA Head and Neck Squamous Cell Carcinomas and GEO databases. Three eligible GEO datasets (GSE65858, GSE41613, and GSE27020) provided data for background adjustment and quantified normalization. Patients without survival information were excluded from further evaluation. In total, 500 HNSCC samples were downloaded from the TCGA database with primary tumors. Ten patients who lacked follow-up information or had a survival time of less than 30 days were excluded from the survival analysis. In addition, masked somatic mutation and copy number variation (CNV) information were also downloaded from the TCGA dataset. The PCD-related genes, which encompassed the fourteen PCD patterns, were gathered from GSEA gene sets and review articles [9,10]. Altogether, 322 intrinsic apoptosis genes, 225 extrinsic apoptosis genes, 148 (MPT)-driven necrosis genes, 36 necroptosis genes, 65 ferroptosis genes, 46 pyroptosis genes, 22 parthanatos genes, 22 entotic cell death genes, 85 netotic cell death genes, 163 lysosome-dependent cell death genes, 456 autophagy genes, 55 mitophagy genes, 35 immunogenic cell death genes and 9 cuproptosis genes were collected for this study. We selected the genes that indicate the poor survival based on the COX analysis.

### ssGSEA and hierarchical clustering analysis

The ssGSEA algorithm was based on specific gene sets of fourteen PCD-related subtypes. We utilized the ssGSEA algorithm with R packages (GSVA, GSEABase, and limma) to comprehensively assess the inherent characteristics of each sample included in the study. To investigate the relationship between PCD-related genes and the clinical phenotype of HNSCC, we utilized the "Consensus Cluster Plus" method with 50 iterations and an 80 % resampling rate. We used the enrichment score of immune items in ssGSEA to cluster the HNSCC samples into distinct groups. The Kaplan-Meier plot was conducted for overall survival (OS) analysis among different clusters.

### ESTIMATE and CIBERSORT analysis

To evaluate the differences in immune response among the three clusters, we employed the "ESTIMATE" package for R (https://sourceforge.net/projects/estimateproject) to calculate the tumor purity, stromal score, immune score, and ESTIMATE score for each HNSCC tumor sample. Then, to further investigate the variations in immune cell subtypes among multiple clusters, we utilized the CIBERSORT package to evaluate the distribution of 22 immune cell types in each sample. The Mann-Whitney U test was used to compare the differences between the two subgroups.

### Calculation of signature from machine learning

To develop a consensus model with high accuracy and stability performance, we integrated 10 machine learning algorithms and 101 algorithm combinations [11]. The integrative algorithms included Random Survival Forest (RSF), Elastic Network (Enet), Lasso, Ridge, Stepwise Cox, CoxBoost, Partial Least Squares Regression for Cox (plsRcox), Supervised Principal Components (SuperPC), Generalized Boosted Regression Modeling (GBM), and Survival Support Vector Machine (Survival-SVM). All models were detected in three datasets (TCGA, GSE65858, GSE41613) with overall survival. For each model, the C-index was calculated across all TCGA and GEO datasets.

### Weighted co-expression network construction

The edgeR R language package was used to identify differentially expressed genes (DEGs) based on the criterion of a *P*-value < 0.05 when comparing across different clusters. Genes with prognostic value and a similar association with cancer were included in the co-expression analysis. To construct the scale-free network, we tested the availability of the prepared DEGs and inputted them into the calculation using the WGCNA R package. First, the appropriate soft threshold power β should be determined to ensure that the network follows a scale-free distribution. Most clinical characteristics were included: sex, race, status, M grade, T grade, N grade, clinical stage, expression of PD-1/PD-L1 and tumor mutation burden (TMB). Next, we converted the expression matrix into the adjacency matrix and applied the business‐linkage hierarchical clustering method to classify genes into different modules using the Dynamic Tree Cut method. A sample clustering tree was constructed, integrating clinical characteristics and co-expression genes, with a minimum requirement of 30 modules for each. Finally, Spearman's correlation coefficient was used to describe the relationship between co-expression modules and clinical factors. Based on the results above, we analyzed the significant correlations between the modules and the immune landscape of HNSCC in order to better identify the crucial module and the hub genes.

### Identification of signaling pathways

Identification of the co-expression module containing functionally similar genes may reveal the major signaling pathways in HNSCC. We, containing functionally similar genes, may reveal the major signaling pathways in HNSCC. We utilized the "clusterProfiler" R package to identify potential biological pathways using the DEGs. GSVA was used to analyze various biological functions among the different groups using the "c5.v7.4.symbols.gmt" database (R packages "GSVA" and "GSEABase").

### Selection of hub genes

Kaplan-Meier survival analyses were performed to further assess the accuracy of the predictions. To enhance the accuracy and interpretability of our predictions, we acquired two GEO cohorts (GSE65858, GSE41613) containing OS data and GSE27020 containing progression-free survival (PFS) data, to serve as validation sets for identifying important prognostic factors.

### Cell lines and cell culture

Tongue cancer cells (Cal27 and HN6) were obtained from ATCC and cultured in Dulbecco's modified Eagle's medium (Procell, Wuhan, China) supplemented with 10 % fetal bovine serum (FBS) (Procell, Wuhan, China). The cells were incubated in a temperature-controlled incubator with 5 % CO_2_.

### RNA interference and transfection

siRNAs and negative controls (NC) were designed and purchased from GenePharma (Suzhou, Jiangsu, China). The sequences of siRNAs targeting FCGR2A (termed si- FCGR2A#1, si- FCGR2A#2, si-FCGR2A#3) are listed in Supplementary Table 1. The knockdown efficiency was determined, and the siRNA with the highest efficiency (termed si-FCGR2A) was selected for further experiments.

### qRT–PCR

Total RNA was extracted from cultured cells using Trizol reagent (Takara). 1 μg of RNA was reverse transcribed into cDNA using a PrimeScript RT Reagent Kit (RR042A, Takara). The relative mRNA expression level was detected by using the 2^−△△CT^ method, with GAPDH acting as the internal loading control. qRT–PCR primer sequences are presented in Table S1.

### CCK8 assays

A total of 3000 cells were seeded into 96-well plates (LABSELECT, China) with 100 μL of medium. Cell proliferation rate was detected using CCK-8 assays (Biosharp, China) at 0, 24, 48, 72, and 96 h. 10 μL of CCK-8 reagent was added to each well at the specified time. After 2 h of incubation at 37 °C, the absorbance was measured at a wavelength of 450 nm.

### Colony formation assay

1000 cells were plated in a 6-well plate with 2 mL of complete medium, and the medium was replaced every 3 days. After 14 days, the colonies were stained with 0.25 % crystal violet.

### Transwell assay

A total of 5 × 10^^4^ cells suspended in serum-free medium were added into the upper chamber, and 600 μL of DMEM containing 20 % FBS was added to the lower chamber. After approximately 24 h of incubation, the cells in the Transwell system were stained with 0.25 % crystal violet. The cells that had migrated across the membrane were imaged and counted.

### Statistical analyses

All statistical analyses in the present study were performed using the R version 4.1.3 software (https://www.r-project.org/), and a *P*-value < 0.05 was considered statistically significant for all the analyses.

## Result

### Assessment of landscape of innate correlation of cell-death types in HNSCC

**Fig. 1:**
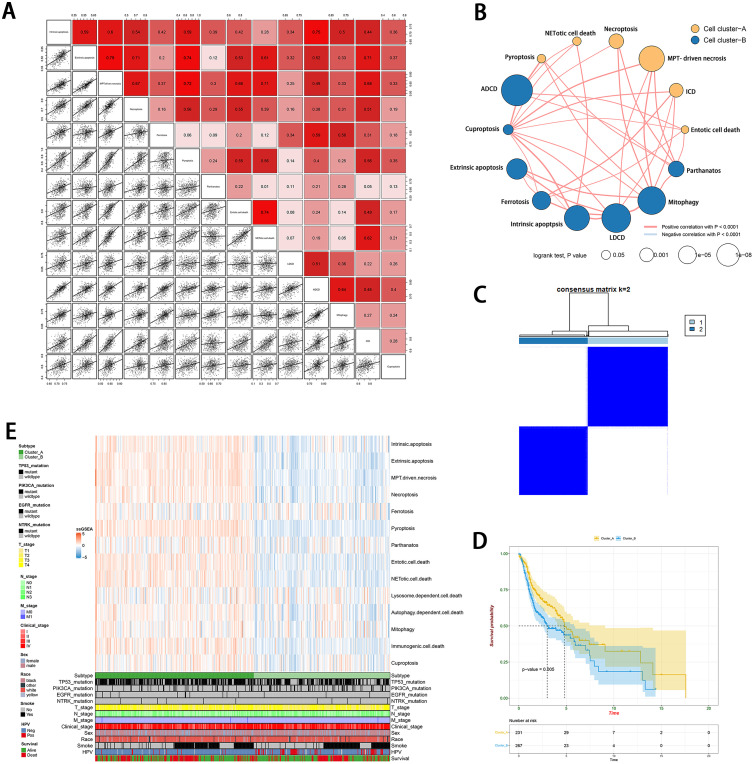
Innate correlation of cell death types in HNSCC. (A) The Spearman correlation analysis was used to evaluate the relationship among 14 types of PCD. (B) The PCD network of cell type interaction based on the correlation strength. (C) Consensus clustering matrix for *k* = 2. (D) Kaplan-Meier curves of OS for two clusters. (E) The heatmap of race, sex, T, N, M stage, gene mutation with high frequency and survival status determined by two clusters.

We planned to identify the genes that indicate poor survival in order to establish a prognostic network based on cell death. Thus, through the COX analysis, we identified 134 PCD-related genes with *P* < 0.05 and HR > 1 for the induce-tumorigenesis cell-death model (Supplementary Tables 2–4). In total, 500 HNSCC samples were obtained from the TCGA database, each with complete clinical characteristics data. Next, the ssGSEA method was applied to the transcriptome of the HNSCC samples to evaluate the scores of 14 cell death types. As shown in Fig. 1A, the Spearman correlation analysis revealed that these cell types in the cancer tissue strongly supported each other. According to the strength of the correlation, these cell types can be divided into two groups. We visualized the PCD network to illustrate a comprehensive landscape of cell interactions, cell lineages, and their effects on each other (Fig. 1B, Supplementary Fig. 1, Supplementary Table 5). Moreover, to gain a deeper understanding of the relationships between these different types of cell death, we conducted a consensus clustering analysis. This analysis aimed to classify the HNSCC samples into two clusters based on the ssGSEA using the Consensus Cluster Plus package with *k* = 2 (Fig. 1C and Supplementary Fig. 2). The Kaplan-Meier method showed that there was a significant difference between clusters A and B (*P* = 0.005) (Fig. 1D). Subsequently, we plotted a heatmap to visualize the relationship among groupings, cell-death scores from ssGSEA, and clinical traits such as HPV, smoke, race, sex, T, N, M stage, and gene mutations with high frequency (Fig. 1E). Furthermore, we observed the remarkable distribution of HPV status and T_Stage (Supplementary Fig. 3).

### Evaluation of tumor-related microenvironment between cell-death subtypes

**Fig. 2:**
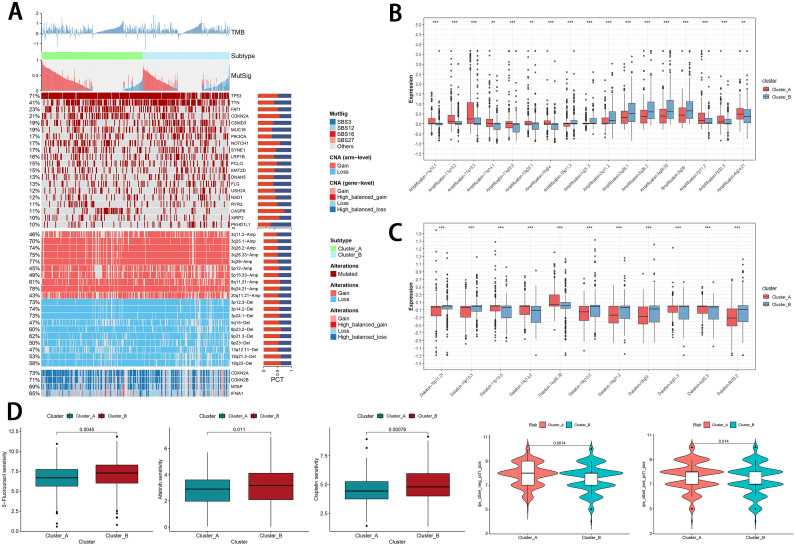
Tumor-related microenvironment between programmed cell death subtypes. (A) Comprehensive heatmap showing the genomic alterations of two cell-death subtypes. (B) The difference in amplification of copy number alterations between two clusters (***P<0.001; **P<0.01; *P<0.05). (C) The difference in deletion of copy number alterations btween two clusters (***P<0.001; **P<0.01; *P<0.05). (D) The meaningful difference in sensitivity of targeted drugs and IPS scores between two clusters.

In Fig. 2A, based on the two clusters [12], we computed the mutational signatures and selected the signatures that were correlated with subtypes (Supplementary Fig. 4A). Of the frequently mutated genes (>5 %), we observed that subtype A harbored more mutations, such as TP53 (*P* = 3.67E^−05^), CASP8 (*P* = 0.000139), and NOTCH1 (*P* = 0.01012). On the other hand, subtype B was enriched with mutations in PKHD1L1 (*P* = 0.017) (Fig. 2A and Supplementary Table 6). In addition, two subtypes exhibited a significant disparity in copy number alterations (CNAs). Especially in regions Ch11q13 and Ch3q26, these regions were found to be susceptible to inactivation during cell immortalization and in various diseases. This may contribute to the development of different subtypes. The results showed that cluster A was enriched in multiple regions when compared to cluster B, including amplification at 11q13.1, 11q13.2, 11q13.3, and 11q13.4. On the other hand, subtype B showed enrichment for amplification at 3q26.2 and 3q26.33 (Fig. 2B). The deletion of CNAs was shown in Fig. 2C. However, we failed to disclose the effect of TMB in HNSCC (Supplementary Fig. 4B), suggesting that HNSCC is heterogeneous cancer regulated by specific cell-death types, not all PCD types. To investigate the influence of the cell-death clusters on anti-tumor therapies, we initially determined the IC50 values of several commonly employed targeted drugs for advanced HNSCC using the oncoPredict package. Subsequently, we predicted the responsiveness of these targeted drugs. For cisplatin, 5-Fluorourail, and Afatinib, the IC50 values of the cluster B were higher, indicating lower sensitivity (Fig. 2D and Supplementary Fig. 5A). In addition, we downloaded the Immune Prognostic Score (IPS) of the TCGA-HNSC cohort from the TCIA database to investigate variations in the effectiveness of immunotherapies between the high- and low-PCD groups. The IPS of ctla4_pos_pd1_pos (a group treating with anti-clta4 and pdl1) and ctla4_neg_pd1_pos was higher in the cluster A (a group treating with anti-pdl1 without anti-ctla4). Interestingly, there was no difference between the IPS of ctla4_pos_pd1_ neg and ctla4_neg_pd1_neg (Fig. 2D and Supplementary Fig. 5B), indicating that patients in the cluster A responded better to anti-PDL1 therapy as well as anti-PD-L1 and anti-CTLA4 combination therapy than those in cluster B.

### Selection of immune-related prognostic cell-death patterns

Preliminary investigations have found that the combination of fourteen cell death pathways cannot accurately predict therapeutic effects and classic clinical characteristics. Therefore, we conducted a new analysis to reassess the relationship between these cell-death types and immune indicators (TMB, PD-L1). As shown in Supplementary Fig. 5A, we found significant correlations between TMB and ferroptosis, immunogenic cell death and NETotic cell death (Supplementary Fig. 6A and Supplementary Fig. 7). Moreover, immunogenic cell death, NETotic cell death, and lysosome-dependent cell death were closely associated with the expression of PD-L1 (Supplementary Fig. 6B and Supplementary Fig. 8). Furthermore, we applied Lasso Cox regression analysis to the 14 cell-death patterns to select specific patterns that are associated with survival (Supplementary Table 7). Patients with HNSCC were divided into low-risk or high-risk groups using the median score as the cutoff. As shown in Supplementary Fig. 6C, we found that as the risk score increased, high-risk patients had significantly worse OS than low-risk patients (*P* < 0.0010). Finally, a total of 11 cell-death patterns were selected to participate in the clinical outcome or immune status. Subsequently, we identified these as important immune-related prognostic types.

### Integrative construction of a consensus signature based on machine learning

**Fig. 3:**
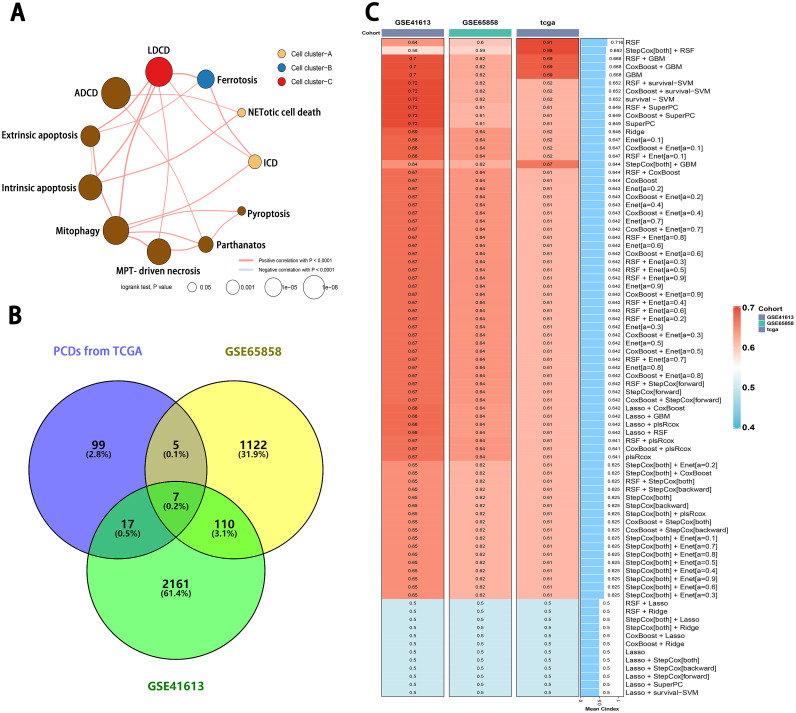
Machine learning-based integrative procedure for testing clinical efficacy. (A) The PCD network of novel cell clusters. (B) The Venn diagram illustrating the overlap of prognostic PCD genes in GSE41613, GSE65858, and TCGA. (C) A total of 101 kinds of prediction models were calculated using the LOOCV framework to determine the C-index actoss all datasets.

Firstly, we presented the correlation network of 11 cell death patterns (Fig. 3A). All patterns can be classified into four cell clusters: TMB-related, PD-L1 related, survival-related, and TMB/PD-L1 related groups (Supplementary Table 8). Then, we applied the COX analysis to calculate the prognostic genes in GSE65858 and GSE41613 (Supplementary Tables 9 and 10). Seven genes were selected based on the interaction of prognostic indicators from three datasets (Fig. 3B). These genes include FCGR2A, CSF2, INHBA, THBS1, FTH1, SERINC3, and TRIM32. Moreover, these genes were subjected to our machine learning-based integrative procedure in order to develop a consensus immune-related cell-death signature. In the TCGA-HNSC dataset, we fitted 101 kinds of prediction models using the LOOCV framework and further calculated the C-index for each model across all datasets (Fig. 3C). Essentially, the optimal model varied across diverse datasets. RSF performed the best on the TCGA datasets, achieving a C-index of 0.91. However, the combination of RSF and survival-SVM yielded the highest average C-index (0.72) for GSE65858, while the Ridge achieved the highest average C-index (0.64) for GSE41613. Overall, the RSF model was found to be the optimal choice for evaluating the prognostic value of cell-death type in HNSC patients.

### Searching for crucial modules through the weighted co-expression network

**Fig. 4:**
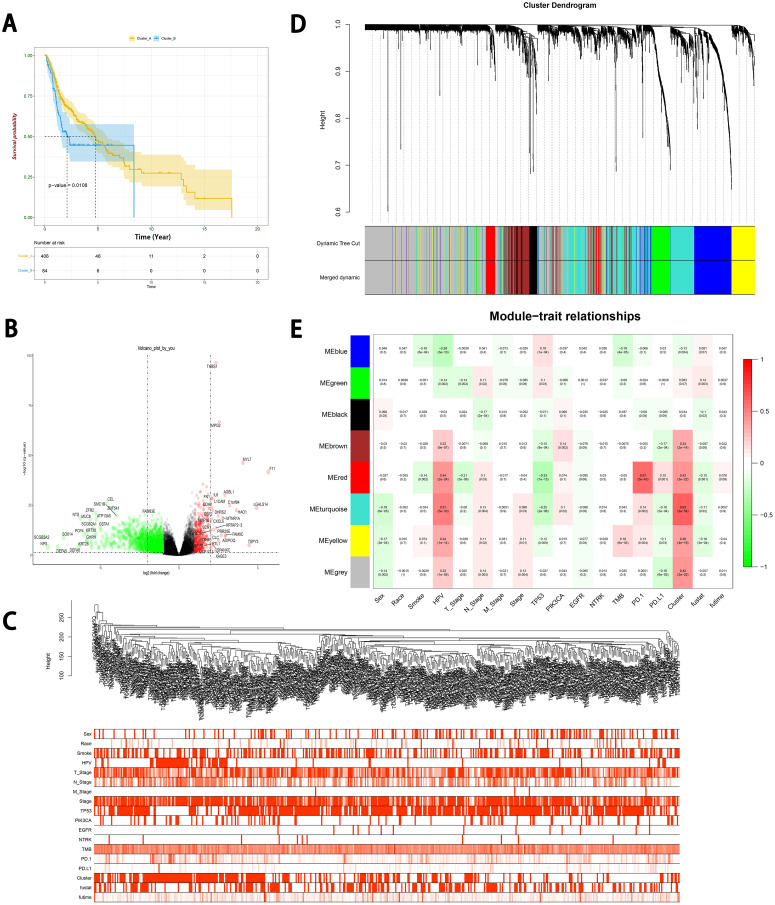
Construction the weighted co-expression network. (A) Kaplan-Meier curves of OS for two clusters based on the 7-PCD gene model. (B) The volcano plot of DEGs from two clusters. (C) Dendrogram and trait heatmap for HNSCC patients. (D) Visualization of a cluster dendrogram through the dynamic tree cut algorithm. (E) Assessing the module-trait relationship of co-expression modules and clinical features.

Based on these 7 regulators, we conducted a consensus clustering analysis to classify them into two clusters using the optimal k value of 2 (Supplementary Fig. 9). The Kaplan-Meier method showed that there was a significant difference between clusters A and B (*P* = 0.0108) (Fig. 4A). Moreover, we utilized the edgeR R language package to identify DEGs. These DEGs were then used to construct a weighted co-expression network, which was visualized in a volcano plot (Fig. 4B). To filter the DEGs, clinical traits such as survival status (live or dead), race, and M, N, and T stages, TMB, multiple mutations, expression of PD-L1/PD-1 were merged. The appropriate soft threshold power β was determined to be six for subsequent adjacency calculations (Supplementary Fig. 10). The dendrogram and trait heatmap for these patients are shown in Fig. 4C and 4D. The data shows that the samples can be divided into multiple groups based on differences in clinical features. In addition, DEGs were classified into eight modules based on their expression. By assessing the relationship between modules and traits, we found that six modules were closely related to cell-death clusters. Furthermore, we observed that the blue module was associated with TMB, while the brown, red, turquoise, yellow, and gray modules were associated with the expression of PD-L1 (Fig. 4E, Supplementary Table 11).

### Exploration the relationship between immune characteristics and PCD modules

**Fig. 5:**
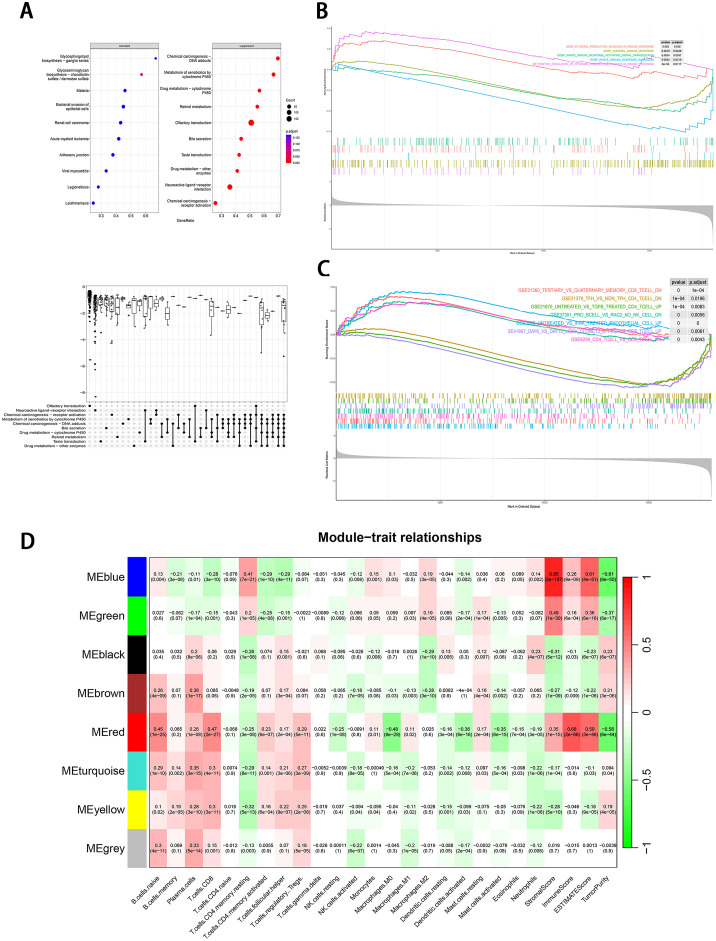
Identification of immune-related PCD modules. (A) The GSEA analysis of DEGs based on the Kyoto Encyclopedia of Genes and Genomes. (B) The GSEA analysis of DEGs based on the MSigDB C5 collection. (C) The GSEA analysis of DEGs based on the MSigDB C7 collection. (D) Visualization of the module-trait relationship using co-expression modules and ESTIMAE, CIBERSORT.

To investigate the potential mechanisms involved in the heterogeneity of HNSCC, we conducted GSEA analysis on the DEGs. The analysis revealed that certain signaling pathways, such as glycosphingolipid biosynthesis and glycosaminoglycan biosynthesis, were activated, while pathways related to chemical carcinogenesis and metabolism of xenobiotics by cytochrome P450 were suppressed during the carcinogenic processes. Additionally, some inhibitory pathways can be co-regulated in HNSCC (Fig. 5A). In the field of immune landscape, we have discovered that specific well-known immune-related pathways were activated in HNSCC through cell-death patterns. These pathways include cytokine production involved in immune response, humoral immune response, and innate immune response (Fig. 5B, Supplementary Figs. 11 and 12). Through the analysis of previous literature on immune pathways, we observed that the DEGs also were significantly associated with the distribution of CD8+, CD4+ *T* cells, TGFβ (Fig. 5C) which involved in the regulation of the immune microenvironment. Based on the above findings, we conducted an ESTIMATE analysis to profile the immune characteristics of HNSCC based on the expression of immune cell types. We used the CIBERSORT method to quantify the level of immune cell infiltration to carefully evaluate the immune landscape. Finally, we evaluated and visualized the relationship between co-expression modules and immune biomarkers. The results showed that this cell-death-based clustering strategy can be used to decipher the heterogeneous immune landscape of HNSCC. We observed that the blue, green, black, brown, red modules were remarkably associated with immune-related scores (immune score and ESTIMATE score). Most modules were related to the distribution of CD4+, CD8+ *T* cells. After integrating modules closely related to TMB or PD-L1, the results showed that blue, brown, and red modules were important modules involved in the immune landscape regulated by specific cell-death genes (Fig. 5D).

### Identification of hub genes in multi-omics analyses

Through the WGCNA analysis, we identified four PCD genes that regulate three modules. Specifically, FCGR2A was found in the blue module, while CSF2, INHBA, and THBS1 were found in the red module. To further identify and verify the predictive ability of four indicators, we downloaded three GEO datasets with OS or progression-free survival (PFS) data and TCGA datasets. In two GEO datasets (GSE41613 and GSE65858) with OS data (Supplementary Fig. 13A and 13B, Supplementary Fig. 14) and TCGA (Supplementary Fig. 13C), three genes were found to be associated with poor OS. However, in GSE27020 dataset with PFS data, only FCGR2A could be re-verified (*P* < 0.0051) (Supplementary Fig. 13D) and considered as the hub gene.

### Interaction and pan-cancer analysis of FCGR2A

**Fig. 6:**
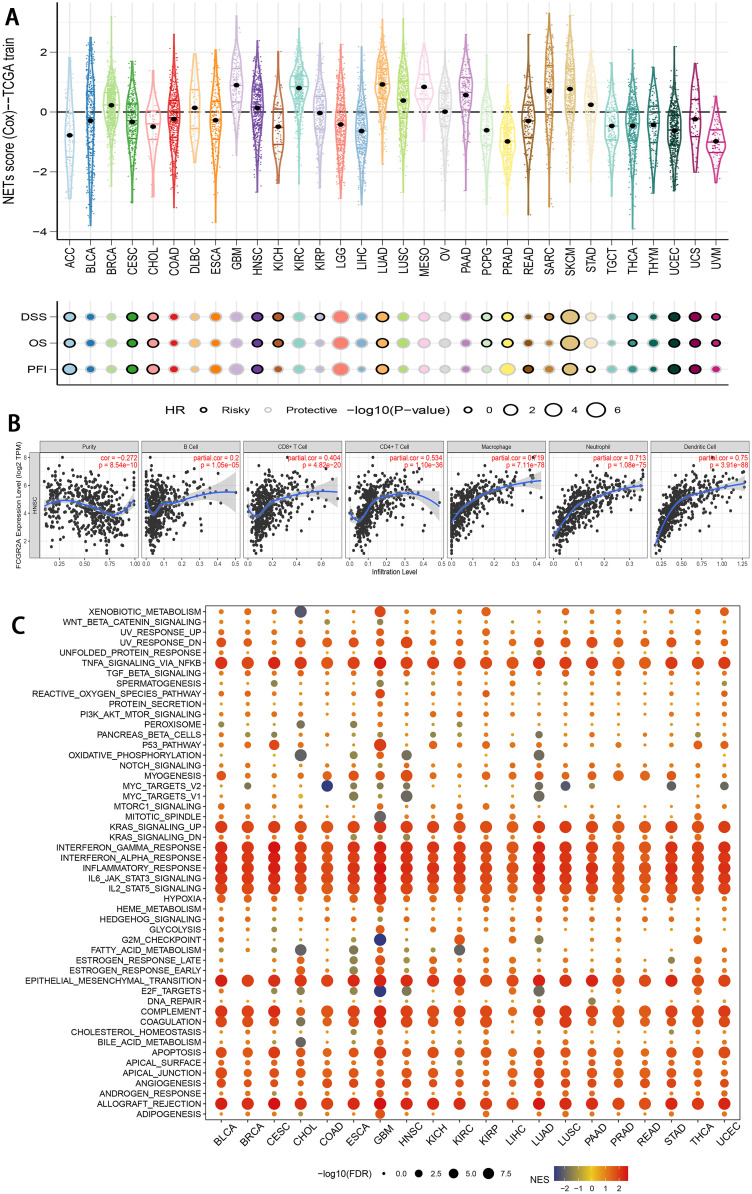
Pan-cancer analysis of FCGR2A. (A) Prognostic performance of the NETosis-related gene, FCGR2A in pan-cancer analysis. (B) The correlation between FCGR2A and immune cell types. (C) The GSEA analysis of FCGR2A based on the Kyoto Encyclopedia of Genes and Genomes pan-cancer analysis.

To determine the crucial molecular relationships of FCGR2A, we conducted a pan-cancer analysis on this oncogene. We downloaded all pan-cancer data from the UCSC Cancer Genomics Browser (https://genome-cancer. ucsc.edu). The results indicated that the expression of FCGR2A was significantly upregulated and acted as a risk factor in most types of cancers, such as lung adenocarcinoma (LUAD) and head and neck squamous cell carcinoma (HNSCC). However, the expression of FCGR2A decreased in bladder urothelial carcinoma (BLCA) and was viewed as having a protective role (Fig. 6A). In HNSCC, FCGR2A was found to be positively correlated with the distribution of CD4+ and CD8+ *T* cells (Fig. 6B). Therefore, we applied the pan-cancer analysis to unravel the principal pathways of FCGR2A and its interaction in the immune landscape. The results indicated that FCGR2A may activate multiple immune genes and interfere with the tumor environment in various types of cancer (Fig. 6C, Supplementary Fig. 15). In this study, we aimed to explore the relevant chemicals involved in the molecular mechanism of FCGR2A. According to the Comparative Toxicogenomics Database, we utilized the molecular docking method to explore potential cancer treatments. These include SM-101 (an FCGR2A inhibitor), cytosine arabinoside, acetaminophen, and Cetuximab with binding energy <−5.0 kcal/mol (Supplementary Fig. 16).

### FCGR2A promotes proliferation, migration and invasion of tongue cancer cells

**Fig. 7:**
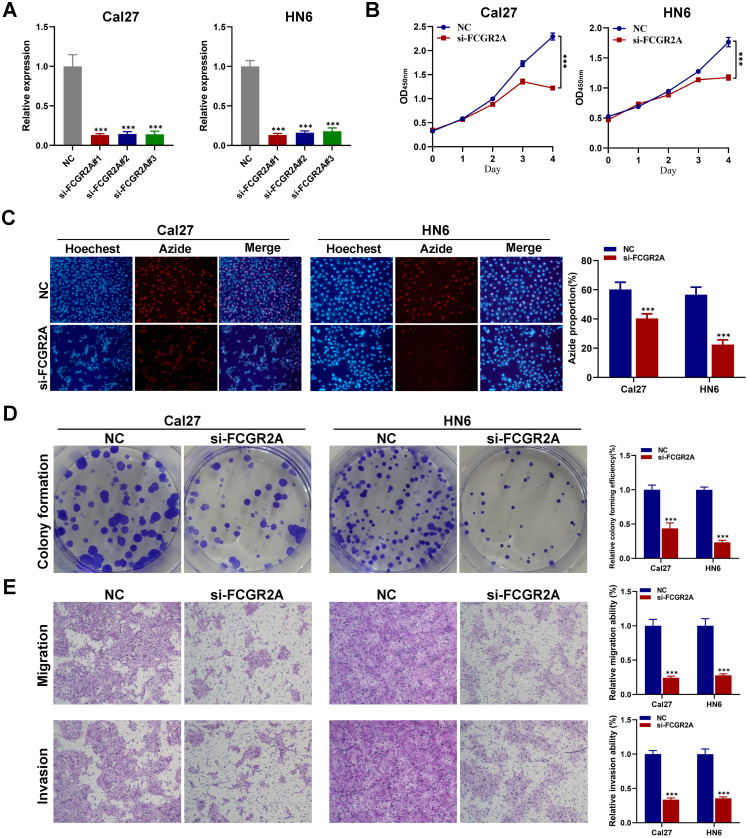
The biological roles of FCGR2A in tongue cancer cells. (A) Verification of FCGR2A knockdown efficiency in Cal27 and HN6 using qRT-PCR. CCK-8 (B), EdU assay (C), colony formation assay (D), and transwell assay (E) were performed to confirm the biological function of FCGR2A in tongue cancer cells. ***P<0.001.

The expression levels of FCGR2A in Cal27 and HN6 cells were reduced by using specific FCGR2A-targeting siRNAs (Fig. 7A). The CCK-8, EdU, and transwell assays verified that knockdown of FCGR2A could inhibit the proliferation, migration, and invasion of Cal27 and HN6 cells (Fig. 7B–E). Thus, these findings reveal that FCGR2A enhances the proliferation, migration, and invasion of HNSC cells.

## Discussion

Globally, HNSCC is the fifth most common cancer and exhibits high levels of heterogeneity and significant differences in therapeutic response [13]. Conventional selection of anti-tumor therapies and clinical management for HNSCC are primarily based on the tumor, lymph node, and metastasis (TNM) classification system. However, for most cases of HNSCC, particularly those in advanced stages, this pre-operative clinical assessment system for tumor and nodal involvement classification often disagrees with treatment responses or prognosis [14]. The variability in clinical outcomes and molecular profiles of this phenomenon has attracted much attention in recent years. Heterogeneous survival represents a significant challenge for oncologists and is considered a critical component of therapeutic response. Cell death is the irreversible cessation of normal tissue function and morphology, as well as the selective elimination of cancer cells without causing harm to normal cells [15]. Tumors are well-known for being notably aggressive biological diseases characterized by an imbalance in cell death. Therefore, triggering alternative programmed cell death is a new therapeutic strategy for HNSCC, especially in cases of chemotherapy and radiotherapy resistance. The Smac analogue birinapant, an inhibitor of IAP, can promote the degradation of the BIRC2 product. This activation of caspase-8-mediated apoptosis and MLKL-mediated necroptosis enhances the sensitivity of radiotherapy in HNSCC [16]. In our study, a total of 134 potential biomarkers were accurately selected for constructing an induce-tumorigenesis cell-death model. Next, we utilized the ssGSEA algorithm to calculate the cell-death scores of each sample in order to depict the tumor environment's regulation of cell death.

It is essential to divide HNSCC patients into two groups based on the cell-death scores. Moreover, in the context of disrupted cell death pathways, we observed significant differences in copy number alterations and gene mutations, including TP53, NOTCH3, and CASP8. To date, the TP53 gene is the most commonly mutated gene, and the mutation rate has significantly increased to 50–70 % in HNSCC [17,18]. HPV-negative HNSCC typically exhibits a TP53 mutation, whereas in HPV-positive cases, TP53 usually remains unmutated and is downregulated by the HPV oncogene E6 [19]. During carcinogenic activation, the TP53 transcription factor is activated, along with CDKN1A, to trigger apoptosis [20]. It also downregulates the mTOR signaling pathway to promote autophagy [21]. In HNSCC, NOTCH1 has been identified as a tumor suppressor gene [22]. In our study, we found that extrinsic apoptosis is a vital pathway correlated with survival and plays a significant role in regulating the immune environment. Interestingly, CASP8 was crucial in propagating the specific extrinsic apoptosis triggered by disruptions in the extracellular microenvironment [23]. It indicates that altering the single nucleotide polymorphism of CASP8 may induce the induce heterogeneous traits of clinical outcomes and immune landscape. In this study, we have identified that Ch3q26 and Ch11q13 were susceptible to inactivation in cell-death types. Similarly, analysis of copy number alterations of HNSCC datasets reveals that gains in the 3q26 region were frequent and seemed to occur later in tumor progression [24]. Furthermore, amplification of 11q13 was found to be significantly associated with decreased PFS and no clinical benefits follwing treatment with a PD-1 inhibitor in a retrospective study conducted in HNSCC patients [25].

In fact, HNSCC is one of the cancer types with the highest level of immune infiltration. TMB, the expression of PD-1 and PD-L1, is the most widely used indicator in immunotherapy indicators to identify clinical beneficiaries [26]. However, the prognostic value of these biomarkers is still unclear because only 20 % of patients would respond to immunotherapies. In our previous study, we conducted ssGSEA to identify immune-related subtypes and observed a significant increase in T cells, CD4 memory-activated T cells, and CD8 T cells within a specific cluster, which was associated with a more favorable prognosis [27]. Moreover, some researchers have found that the levels of immune cell infiltration, including the distribution of CD8+ *T* cells in the tumor microenvironment, can predict the clinical efficacy of PD1 inhibitors more accurately than the expression of PD1 or PD-L1 or TMB [28].

Compared to the other approaches, WGCNA showed improved predictive ability and effectiveness in identifying genes with similar expression patterns. It also facilitated the visualization of relationship between PCD genes and clinical features [29]. In our study, we applyed the WGCNA to accurately select numerous genes associated with PCD that are correlated with immune-related clinical parameters (TMB, PD1 or PD-L1), and immune infiltrating characteristics including CD8+ and CD4+ *T* cells. Finally, three co-expression modules, namely blue, brown, and red, were identified to modify the intricate immune landscape in HNSCC.

In the meantime, we also conducted comprehensive testing on the clinical predictive efficacy of the aforementioned PCD genes. In this study, we employed an integrative approach that utilized a range of machine learning algorithms and their combinations. This procedure aims to reduce the dimensionality of variables and make the model more accurate in predicting the prognosis of HNSCC. In a total, 101 kinds of models were fitted to the HNSCC dataset using the LOOCV framework. Eventually, the prognostic meta-analysis demonstrated that the cell-death model was a deleterious indicator with great accuracy and stable performance. Additionally, we selected a GEO dataset with PFS to validate the hub genes. Only FCGR2A was successfully re-verified which was a great role in regulation of PCD not depending on HPV status, T, N, M stage and primary sites (Supplementary Figs. 17 and 18).

FCGR2A, a member of the immunoglobulin Fc receptor family and the NETosis pathway, has been identified on the surface of various immune response cells [30]. In some studies, FCGR2A-rs1801274 was found to be associated with an elevated risk of lung cancer [31]. Furthermore, Holgado et al. revealed that FCGR2A could promote the activation of CD4+ *T* cells [32]. In our study, we identified FCGR2A as a core gene to directly interact with immune landscape, promoting the activation of cell-death patterns into different anti-tumor profiles. In addition, we also identified two hub genes, THBS1 and CSF2, which were successfully re-verified in all GEO datasets with OS. THBS1, an adhesive glycoprotein that mediates cell-to-cell and cell-to-matrix interactions, was essential to be the induction of apoptosis. Xiao M et al. proposed a novel paracrine loop between cancer cells and macrophages that exosome-transferred THBS1 can activate specific M1-like tumor-associated macrophages, promoting malignant migration in oral squamous cell carcinoma [33]. CSF2 is a cytokine functioning as hematologic cell growth factor. Yiming Li et al. reported that the exposure to exogenous CSF2 can promote the alternative macrophage transition to attenuate the release of the apoptosis and reactive oxygen species (ROS) in acute kidney injury [34]. Furthermore, our study is the first to depict these genes altering the tumor immune environment in regulation of PCD.

None of the past studies focused on the hole cell-death types in HNSCC. Thus, our study comprehensively unraveled the genomic properties of programmed cell death in the field of immunology. Our results indicate that PCD genes are promising predictive indicators that offer new insights into effective immunotherapy. Certainly, further clinical trials and molecular experiments are required to verify our results.

## Conclusion

Our study comprehensively analyzed all PCD in HNSCC and strongly supports their regulatory role in immune characteristics. Our results indicate that specific PCD genes are promising targets for effective immunotherapy.

## Data availability statement

Publicly available datasets were analyzed in this study. This data can be found here: The raw data and corresponding clinical information were downloaded from the Genomic Data Commons (GDC, https://portal.gdc.cancer.gov/) and Gene Expression Omnibus Database (GEO; https://www.ncbi.n lm.nih.gov/geo/).

## Ethics statement

The study was conducted in accordance with the Declaration of Helsinki. Ethical approval was obtained at all participating sites, and all the participants provided signed, written, informed consent.

Supplementary Fig. 1. The classification of ssGSEA scores based on correlation strength.

## Funding sources

This work was supported by the Key Laboratory of Translational Radiation Oncology, Hunan Province (No. 2015TP1009), the Provincial Key Research and Development Program of Hunan Province (No. 2018SK2123), Hunan Cancer Hospital Climb Plan (No. QH201905), Scientific Research Project of Hunan Provincial Health Commission (B202309037920), Scientific Research Fund of Hunan Administration of traditional Chinese medicine (B2023007), Hunan Provincial Natural Science Foundation of China (2023JJ40407).

## CRediT authorship contribution statement

**Yi Jin:** Data curation, Formal analysis, Funding acquisition, Visualization, Writing – original draft. **Siwei Huang:** Writing – original draft, Writing – review & editing. **Hongyu Zhou:** Validation. **Zhanwang Wang:** Methodology, Validation. **Yonghong Zhou:** Writing – review & editing.

## Declaration of competing interest

The authors declare that they have no known competing financial interests or personal relationships that could have appeared to influence the work reported in this paper.

## Appendix

HNSCC Head and neck squamous cell carcinoma

PCD Programmed cell death

EGFR Epidermal growth factor receptor

TCGA The Cancer Genome Atlas ssGSEA Single-sample gene set enrichment analysis

NCCD The Nomenclature Committee on Cell Death

MPT Mitochondrial permeability transition

OS The overall survival

RSF Random survival forest

Enet Elastic network plsRcox Partial least squares regression for Cox

SuperPC Supervised principal components

GBM Generalised boosted regression modeling

Survival-SVM Survival support vector machine

DEG Differentially expressed gene

CNA Copy number alteration

TMB The tumor burden

PFS Progression-free survival

LUAD Lung adenocarcinoma

BLCA Bladder urothelial carcinoma

TNM Tumor, lymph node, and metastasis

ROS Reactive oxygen species

## Appendix B Supplementary materials

Supplementary Fig. 2. Consensus clustering matrix was generated for values of k ranging from 1 to 9. In addition, the consensus clustering cumulative distribution function (CDF) and the relative change in the area under the CDF curve were calculated. Image, image 1

Supplementary Fig. 3. Different distribution of clinical features in two PCD clusters. Image, image 2

Supplementary Fig. 4. Evaluation of mutation signatures and TMB. (A) Different expressions of meaningful mutation signatures in two PCD clusters. (B) Different expressions of TMB in two PCD clusters. Image, image 3

Supplementary Fig. 5. The sensitivity of targeted drugs and IPS scores between two clusters. Image, image 4

Supplementary Fig. 6. Selection of immune-related PCD patterns. (A) The relationship between TMB and specific PCD (ferroptosis, immunogenic cell death and NETotic cell death) with *P*<0.05. (B) The relationship between PD-L1 and specific PCD (immunogenic cell death, lysosome-dependent cell death and NETotic cell death) with *P*<0.05. (C) Overall survival analysis for high/low risk groups in LASSO analysis. Image, image 5

Supplementary Fig. 7. The relationship between TMB and other PCDs. Image, image 6

Supplementary Fig. 8. The relationship between PD-L1 and other PCDs. Image, image 7

Supplementary Fig. 9. Consensus clustering matrix for *k* = 2 to 9 basen on 7 regulators model. Image, image 8

Supplementary Fig. 10. Parameters of the construction in WGCNA. (A) Visualization of check scale free topology. (B) Network topology obtained by the soft-threshold power analysis method. (C) A heatmap describing the TOM between random genes from the network. Image, image 9

Supplementary Fig. 11. The visualization of all immune-related pathways based on the MSigDB C5 collection by the GSEA analysis. Image, image 10

Supplementary Fig. 12. The visualization of all immune-related pathways based on the MSigDB C7 collection was done using the GSEA analysis. Image, image 11

Supplementary Fig. 13. Hub genes selection and validation based on four PCD genes. (A) Kaplan-Meier curves for patients in GSE41613 with OS. (B) Kaplan-Meier curves for patients in GSE65858 with OS. (C) Kaplan-Meier curves for patients in TCGA with OS. (D) Kaplan-Meier curves for patients in GSE27020 with PFS. Image, image 12

Supplementary Fig. 14. Evaluation of the predictive value of 3 PCD genes (FTH1, SERINC3, and TRIM32). (A) Kaplan-Meier curves for patients in GSE27020 with PFS. (B) Kaplan-Meier curves for patients in GSE41613 with OS. (C) Kaplan-Meier curves for patients in GSE65858 with OS. (D) Kaplan-Meier curves for patients in TCGA with OS. Image, image 13

Supplementary Fig. 15. The GSEA analysis of FCGR2A based on the MSigDB C5 collection and Genomes pan-cancer analysis. Image, image 14

Supplementary Fig. 16. Exploration of possibility treatment in molecular docking method. (A) Molecular docking of FCGR2A and SM-101. (B) Molecular docking of FCGR2A and Cytosine arabinoside. (C) Molecular docking of FCGR2A and Acetaminophen. (D) Molecular docking of FCGR2A and Cetuximab. Image, image 15

Supplementary Fig. 17. The relationship between FCGR2A and clinical features. Image, image 16

Supplementary Fig. 18. The expression of FCGR2A in all the primary sites of HNSCC. Image, image 17

Supplementary Table 1. The sequences of primers and siRNAs used in the research. Image, image 18

Supplementary Table 2. The list of all genes from 14 PCD types. Image, application 19

Supplementary Table 3. The prognostic genes in TCGA HNSCC dataset with *P* < 0.05 and HR >1 by the Cox analysis. Image, application 20

Supplementary Table 4. The list of 134 PCD-related genes for the induce-tumorigenesis cell-death model. Image, application 21

Supplementary Table 5. The Cox analysis of 14 PCD scores from the ssGSEA method. Image, application 22

Supplementary Table 6. The list of gene mutation with high frequency. Image, application 23

Supplementary Table 7. Lasso Cox regression analysis of 14 PCD types. Image, application 24

Supplementary Table 8. A novel cell cluster of selected 11 types of PCD. Image, application 25

Supplementary Table 9. The Cox analysis in GSE65858. Image, application 26

Supplementary Table 10. The Cox analysis in GSE41613. Image, application 27

Supplementary Table 11. The list of DEGs in every module from WGCNA. Image, application 28

Image, application 29
