# Supplementary figures and images for: Multi-omics comprehensive analyses of programmed cell death patterns to regulate the immune characteristics of head and neck squamous cell carcinoma
Source: Transl Oncol. 2024 Jan 18;41:101862. doi: 10.1016/j.tranon.2023.101862 (PMC10825548; doi:10.1016/j.tranon.2023.101862)

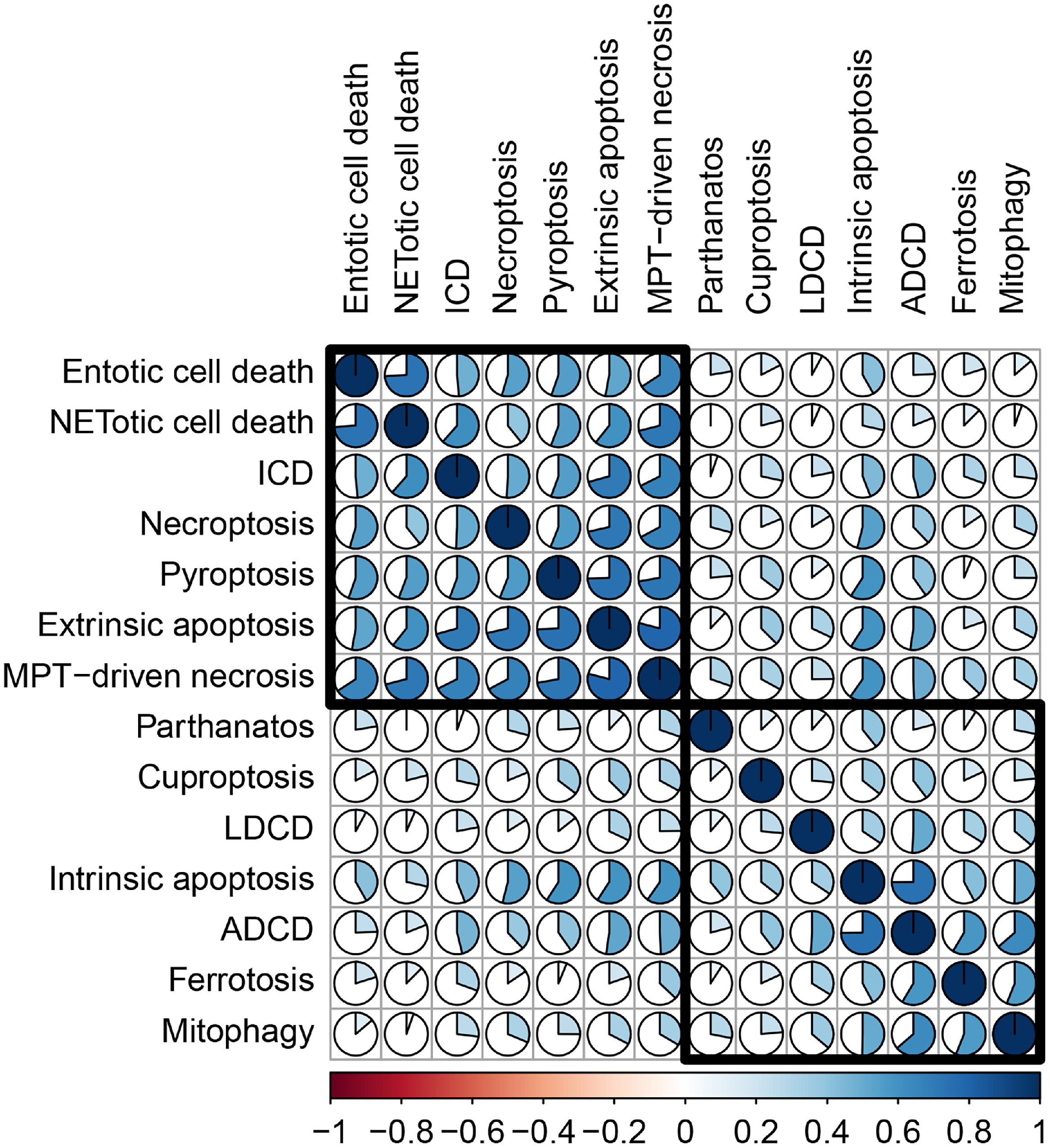

Supplement: Supplementary file 1 — Supplementary Fig. 2. Consensus clustering matrix was generated for values of k ranging from 1 to 9. In addition, the consensus clustering cumulative distribution function (CDF) and the relative change in the area under the CDF curve were calculated. [file mmc1.jpg]

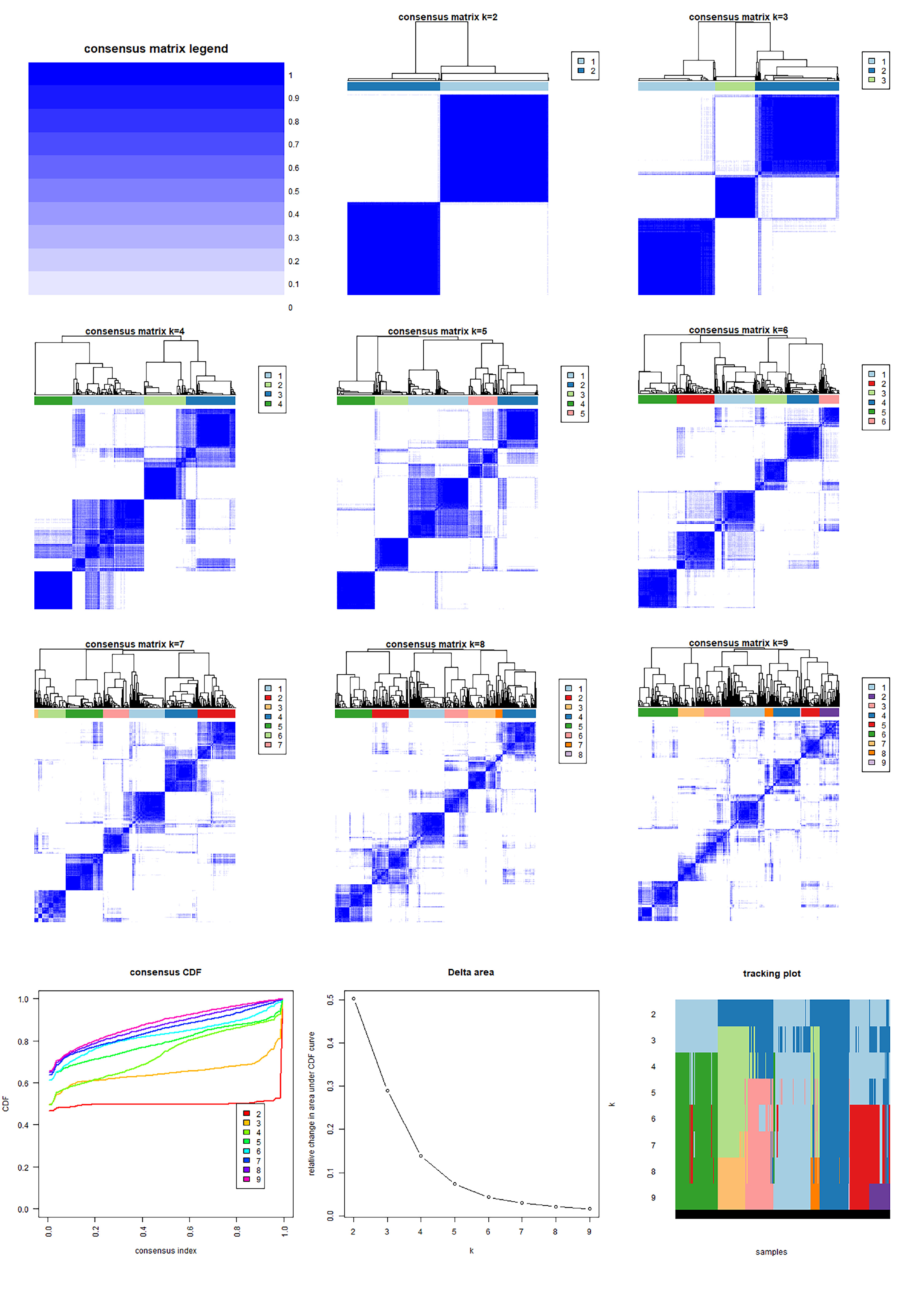

Supplement: Supplementary file 2 — Supplementary Fig. 3. Different distribution of clinical features in two PCD clusters. [file mmc2.jpg]

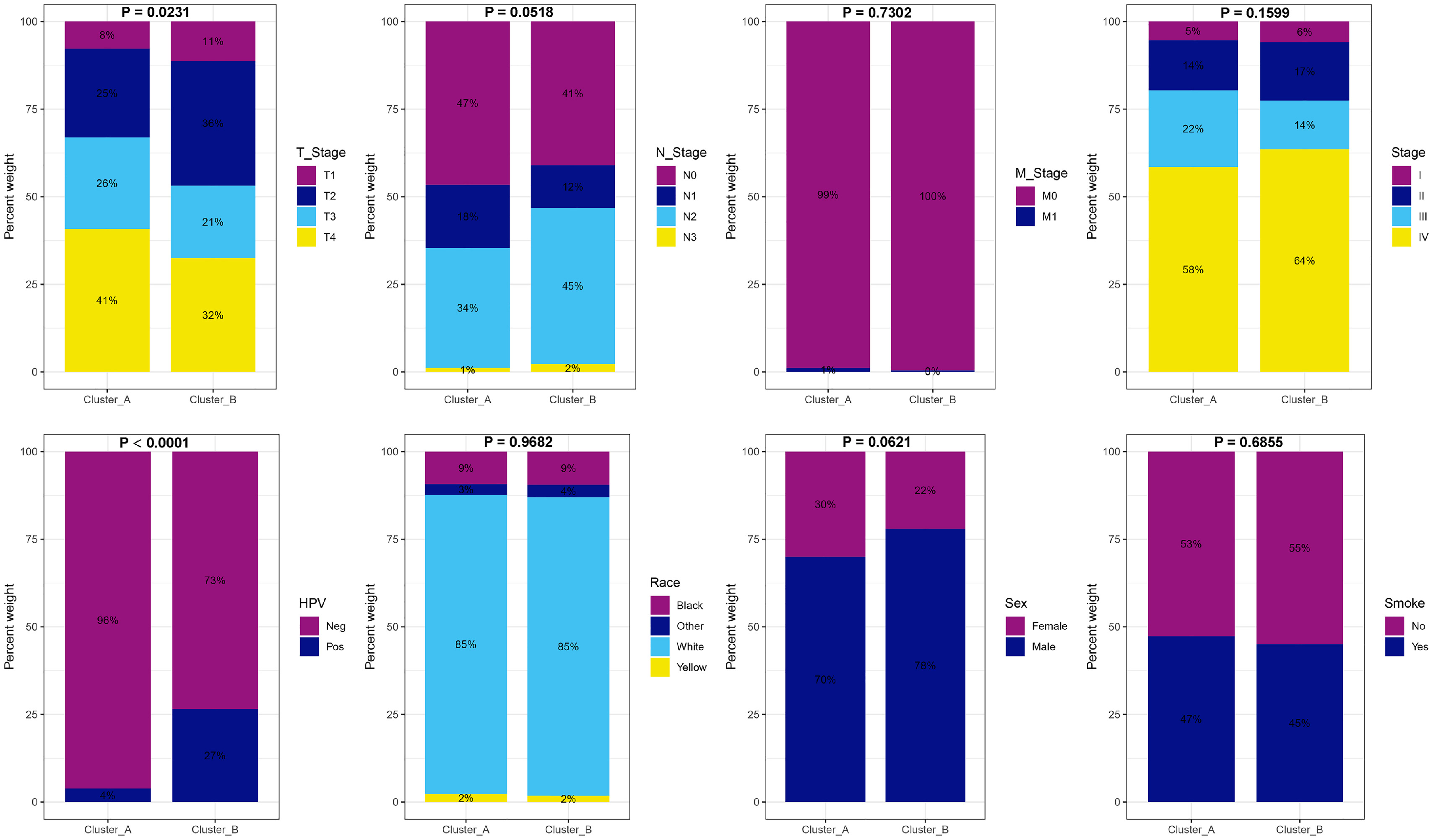

Supplement: Supplementary file 3 — Supplementary Fig. 4. Evaluation of mutation signatures and TMB. (A) Different expressions of meaningful mutation signatures in two PCD clusters. (B) Different expressions of TMB in two PCD clusters. [file mmc3.jpg]

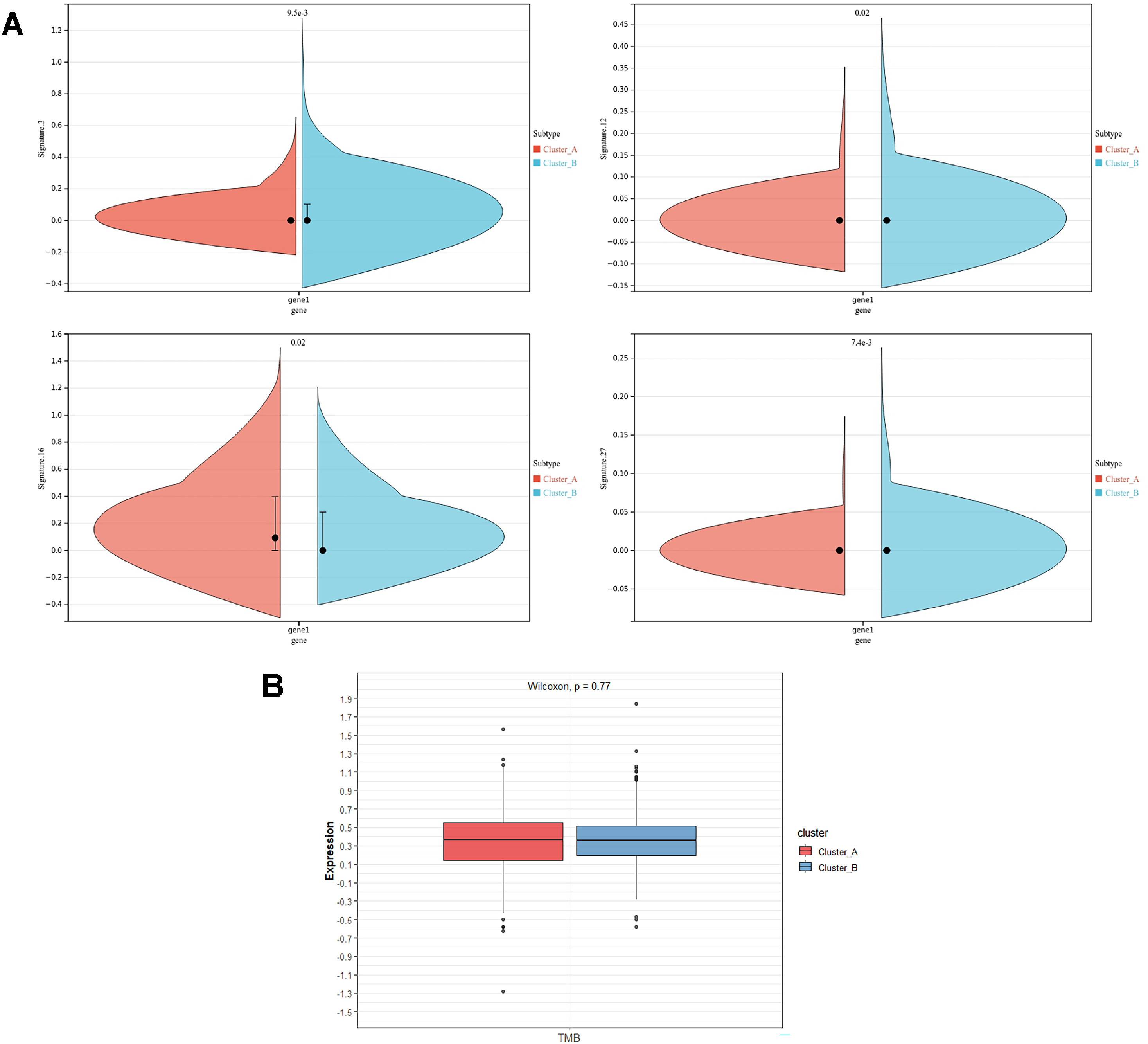

Supplement: Supplementary file 4 — Supplementary Fig. 5. The sensitivity of targeted drugs and IPS scores between two clusters. [file mmc4.jpg]

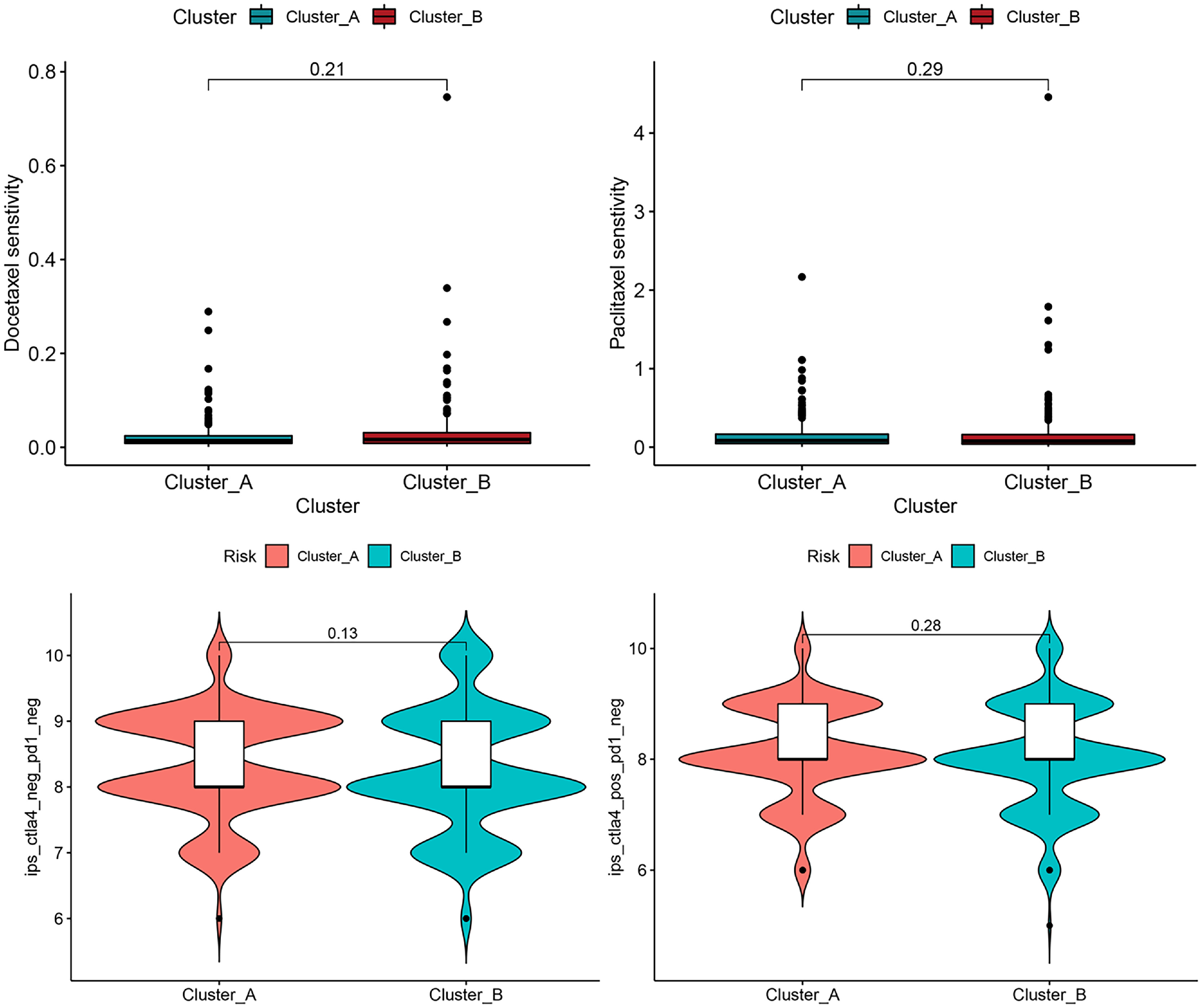

Supplement: Supplementary file 5 — Supplementary Fig. 6. Selection of immune-related PCD patterns. (A) The relationship between TMB and specific PCD (ferroptosis, immunogenic cell death and NETotic cell death) with P<0.05. (B) The relationship between PD-L1 and specific PCD (immunogenic cell death, lysosome-dependent cell death and NETotic cell death) with P<0.05. (C) Overall survival analysis for high/low risk groups in LASSO analysis. [file mmc5.jpg]

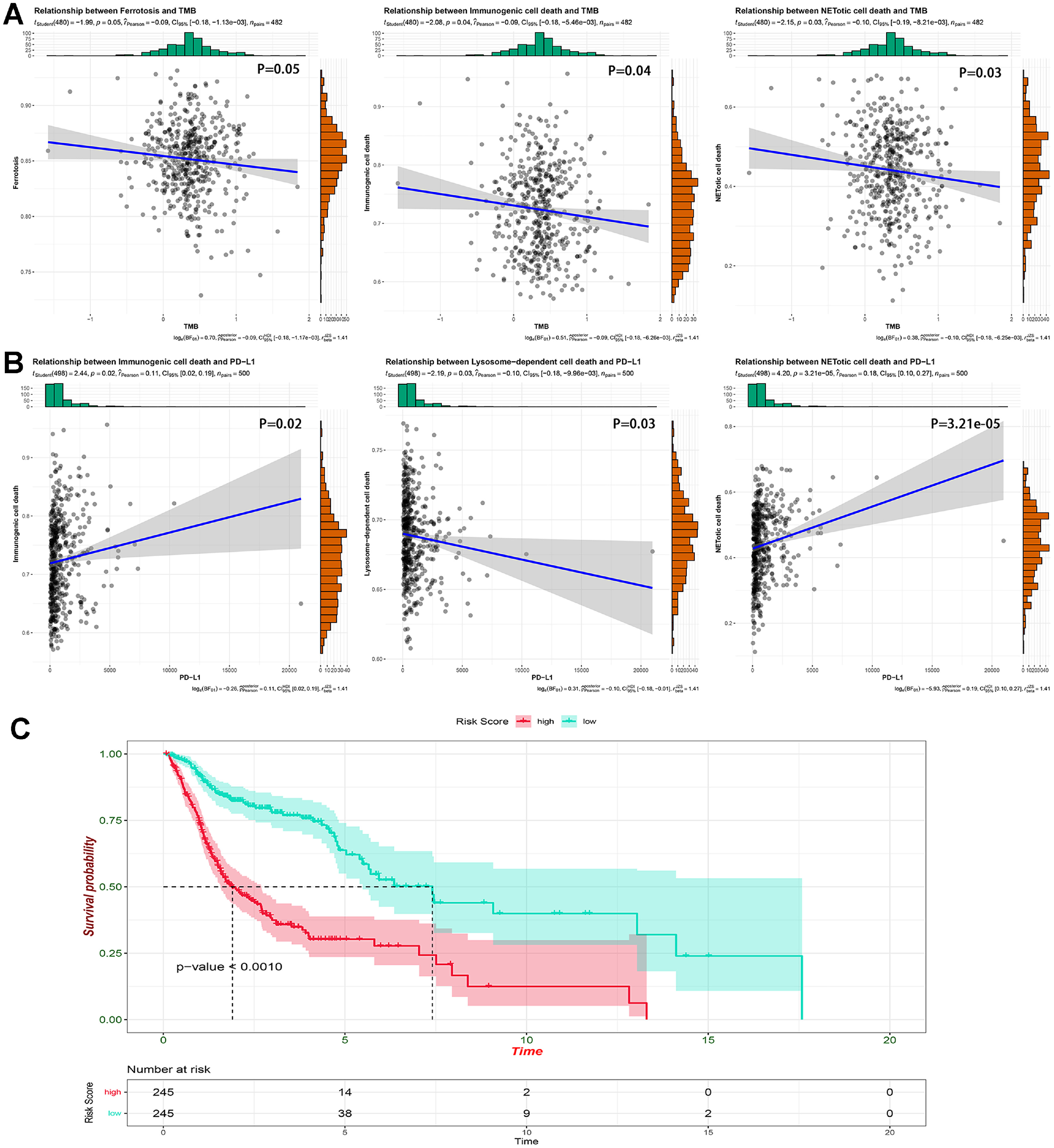

Supplement: Supplementary file 6 — Supplementary Fig. 7. The relationship between TMB and other PCDs. [file mmc6.jpg]

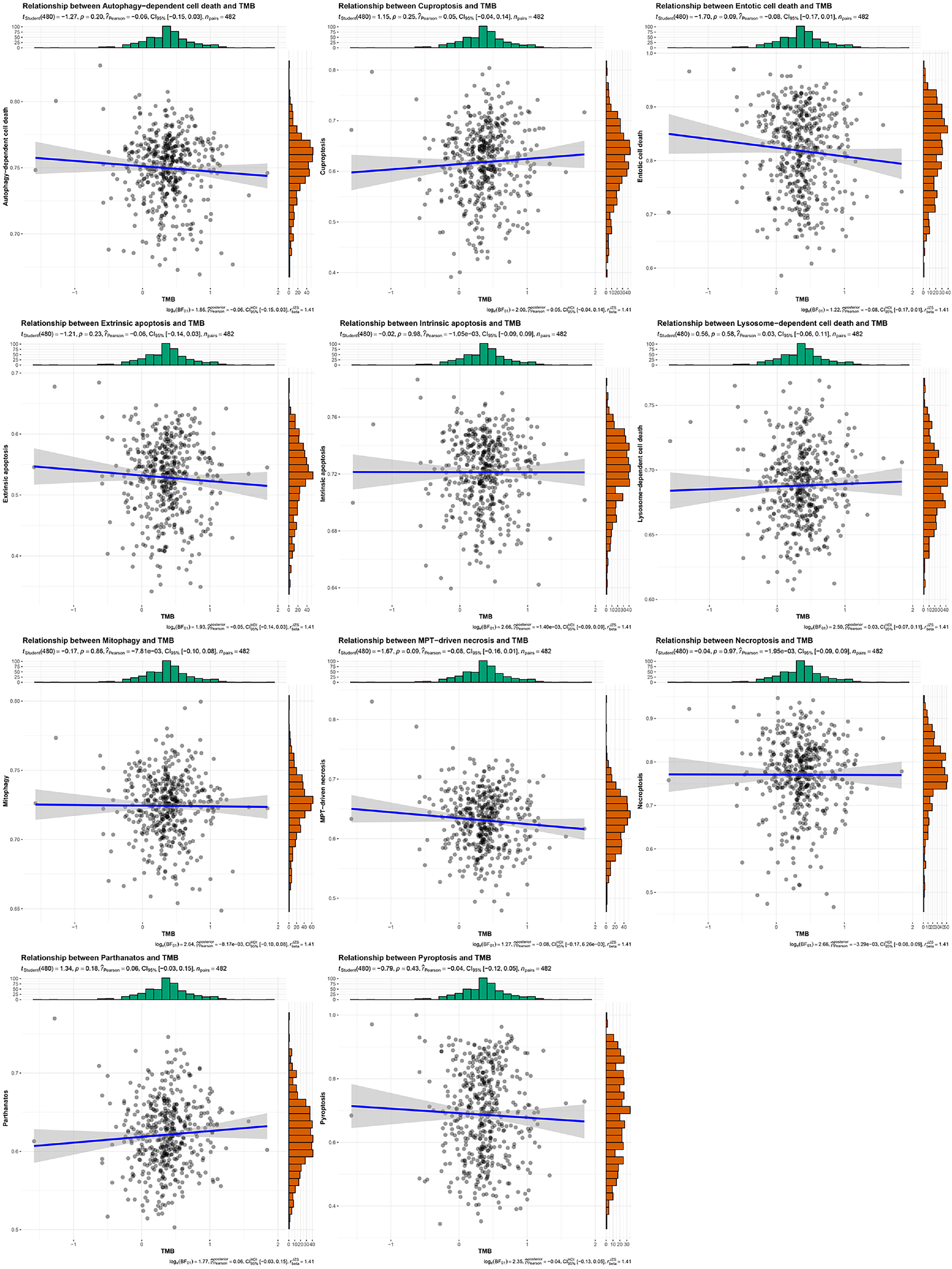

Supplement: Supplementary file 7 — Supplementary Fig. 8. The relationship between PD-L1 and other PCDs. [file mmc7.jpg]

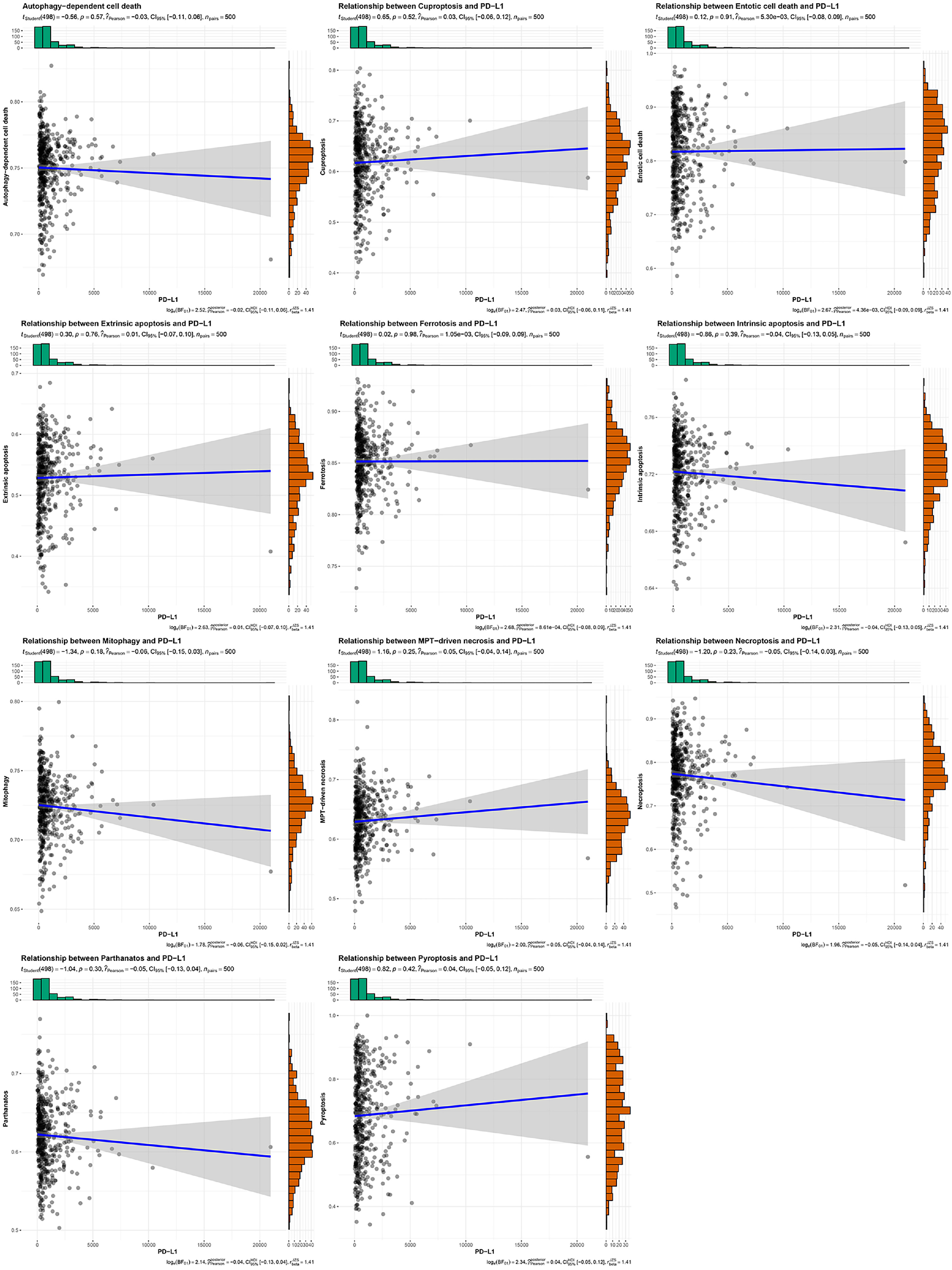

Supplement: Supplementary file 8 — Supplementary Fig. 9. Consensus clustering matrix for k = 2 to 9 basen on 7 regulators model. [file mmc8.jpg]

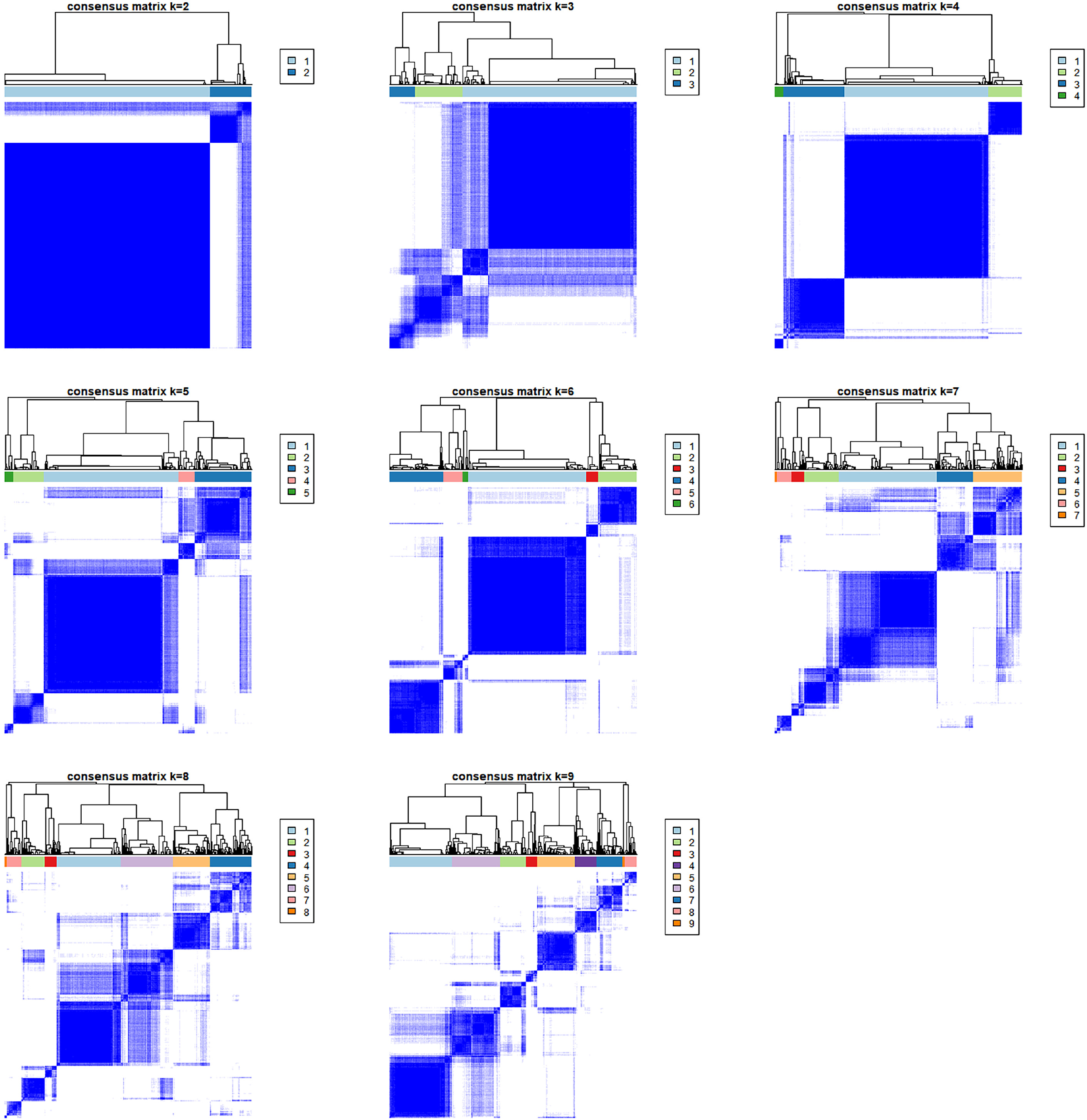

Supplement: Supplementary file 9 — Supplementary Fig. 10. Parameters of the construction in WGCNA. (A) Visualization of check scale free topology. (B) Network topology obtained by the soft-threshold power analysis method. (C) A heatmap describing the TOM between random genes from the network. [file mmc9.jpg]

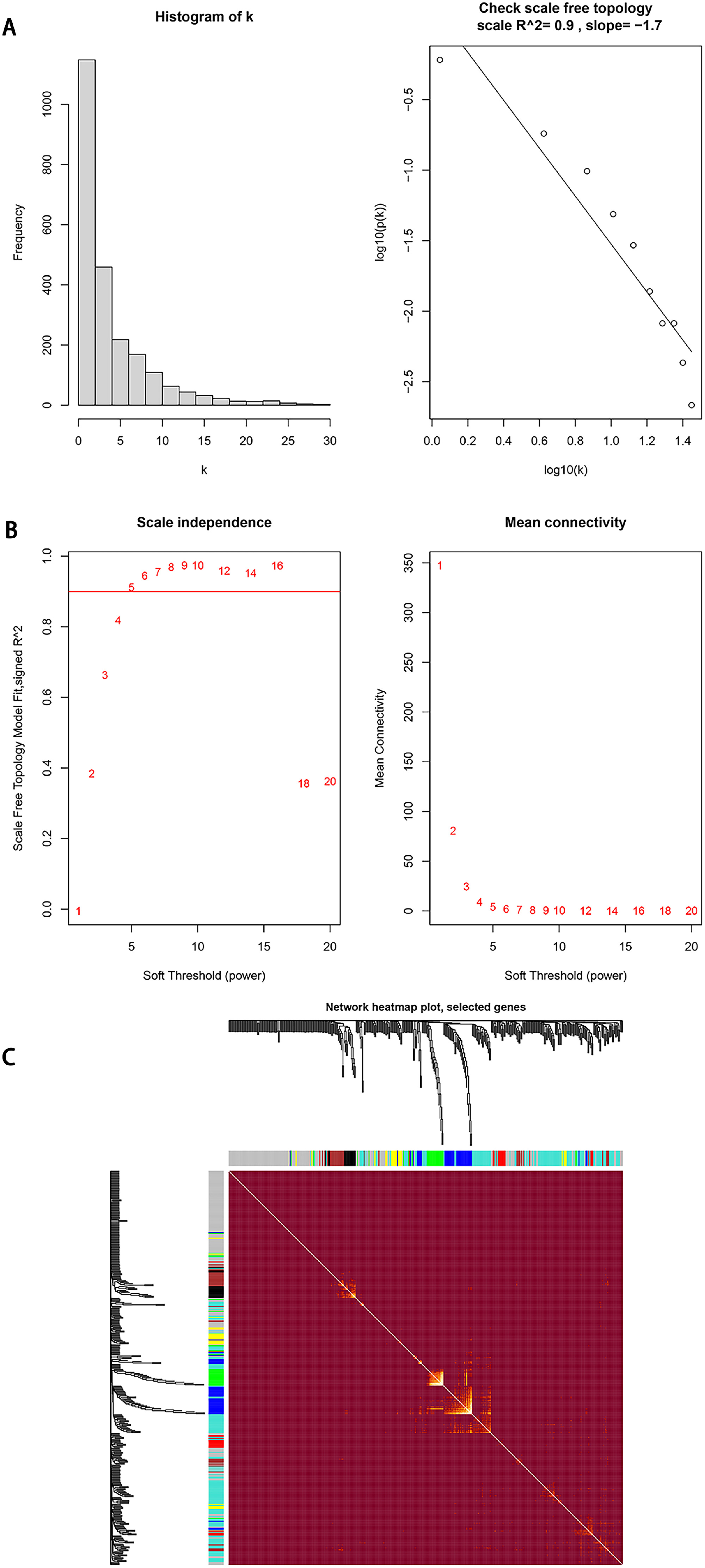

Supplement: Supplementary file 10 — Supplementary Fig. 11. The visualization of all immune-related pathways based on the MSigDB C5 collection by the GSEA analysis. [file mmc10.jpg]

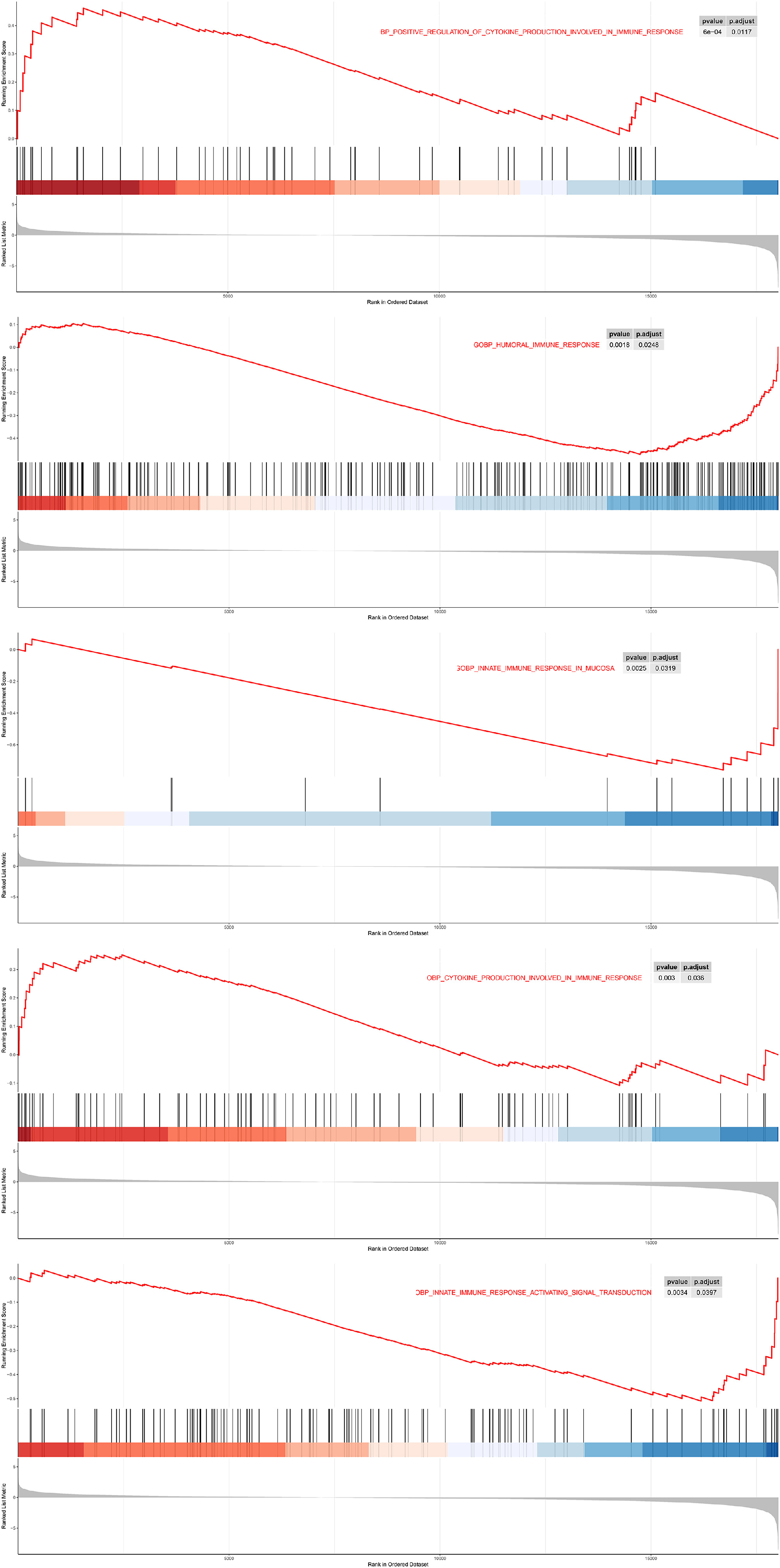

Supplement: Supplementary file 11 — Supplementary Fig. 12. The visualization of all immune-related pathways based on the MSigDB C7 collection was done using the GSEA analysis. [file mmc11.jpg]

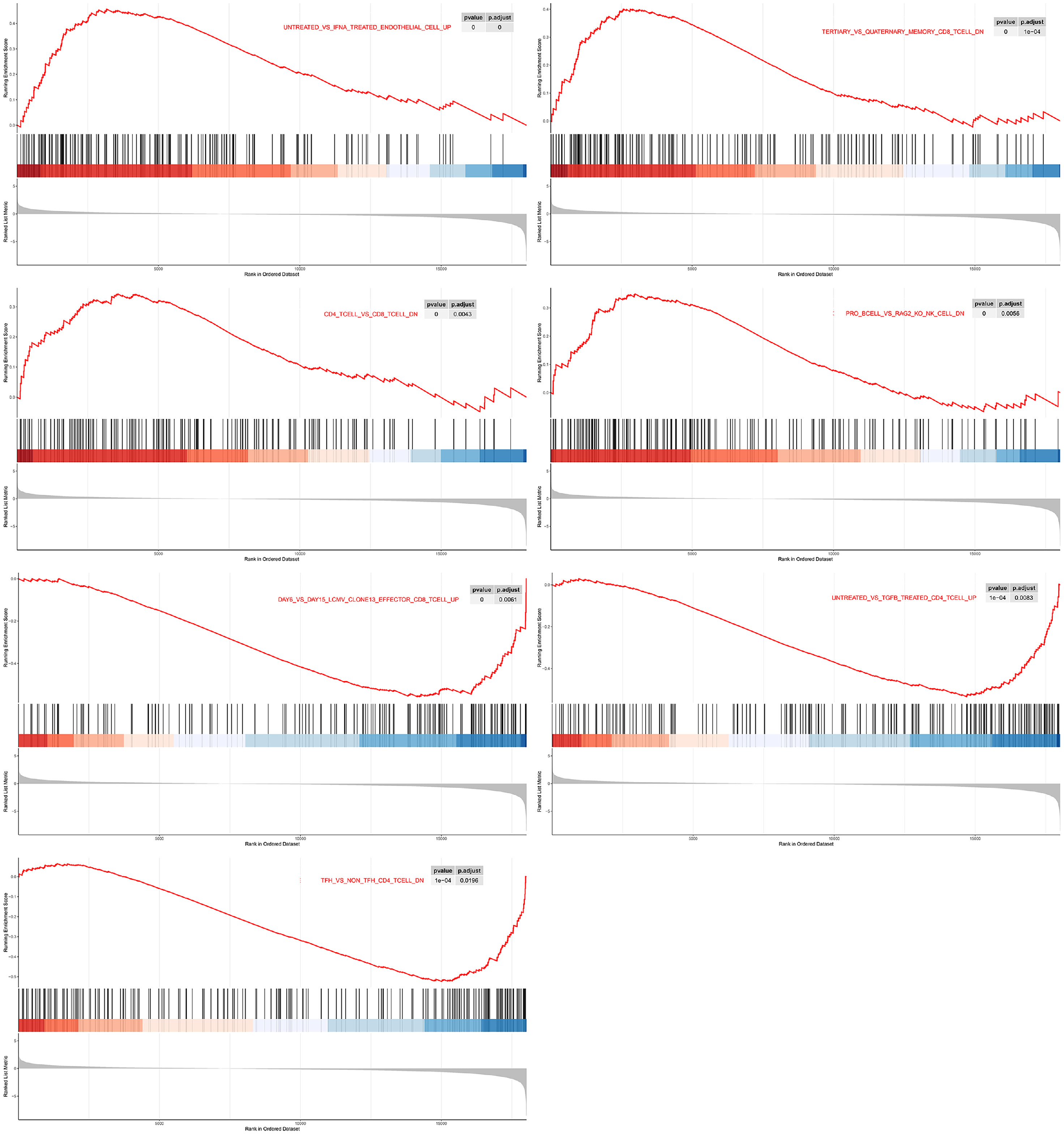

Supplement: Supplementary file 12 — Supplementary Fig. 13. Hub genes selection and validation based on four PCD genes. (A) Kaplan-Meier curves for patients in GSE41613 with OS. (B) Kaplan-Meier curves for patients in GSE65858 with OS. (C) Kaplan-Meier curves for patients in TCGA with OS. (D) Kaplan-Meier curves for patients in GSE27020 with PFS. [file mmc12.jpg]

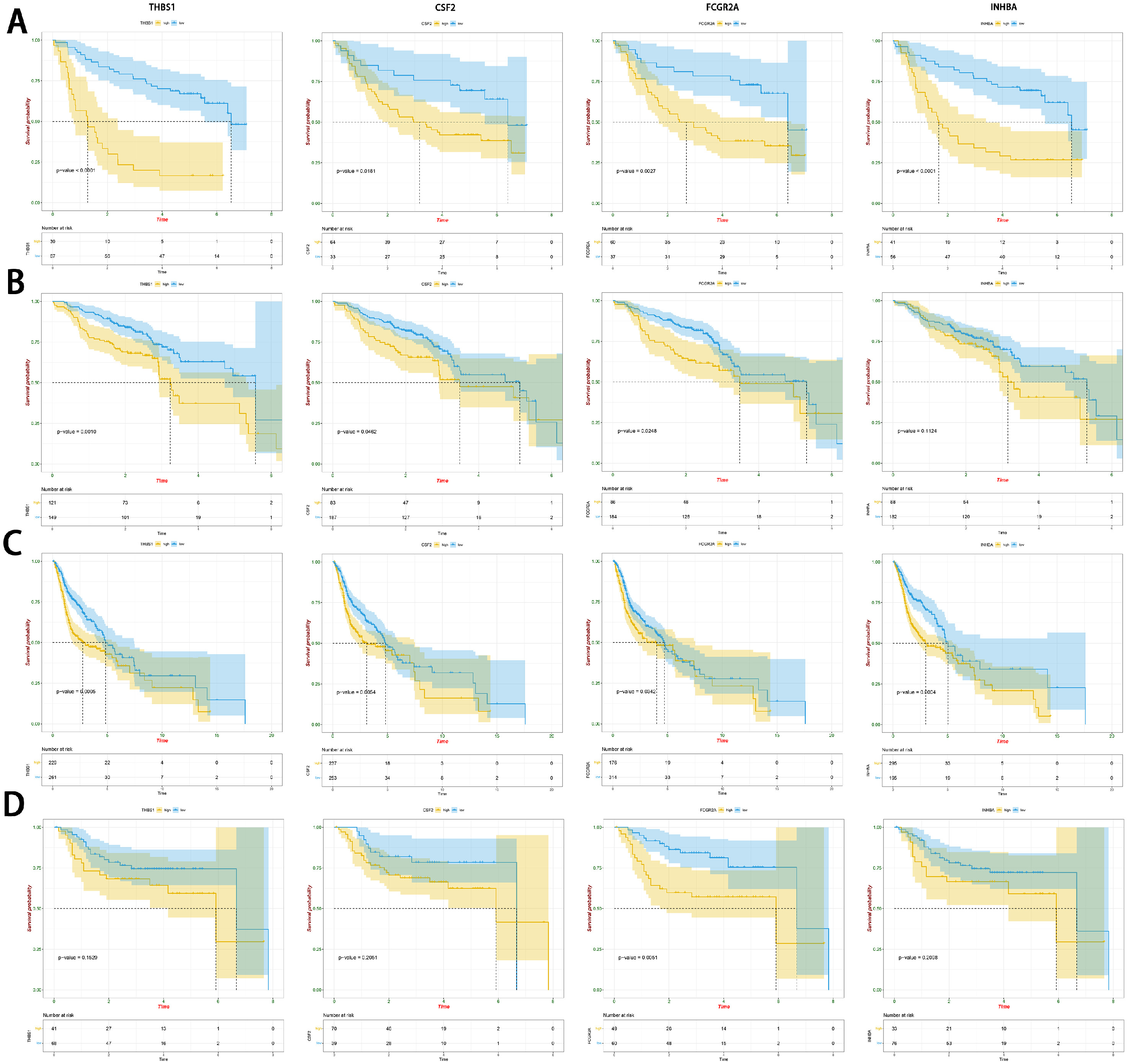

Supplement: Supplementary file 13 — Supplementary Fig. 14. Evaluation of the predictive value of 3 PCD genes (FTH1, SERINC3, and TRIM32). (A) Kaplan-Meier curves for patients in GSE27020 with PFS. (B) Kaplan-Meier curves for patients in GSE41613 with OS. (C) Kaplan-Meier curves for patients in GSE65858 with OS. (D) Kaplan-Meier curves for patients in TCGA with OS. [file mmc13.jpg]

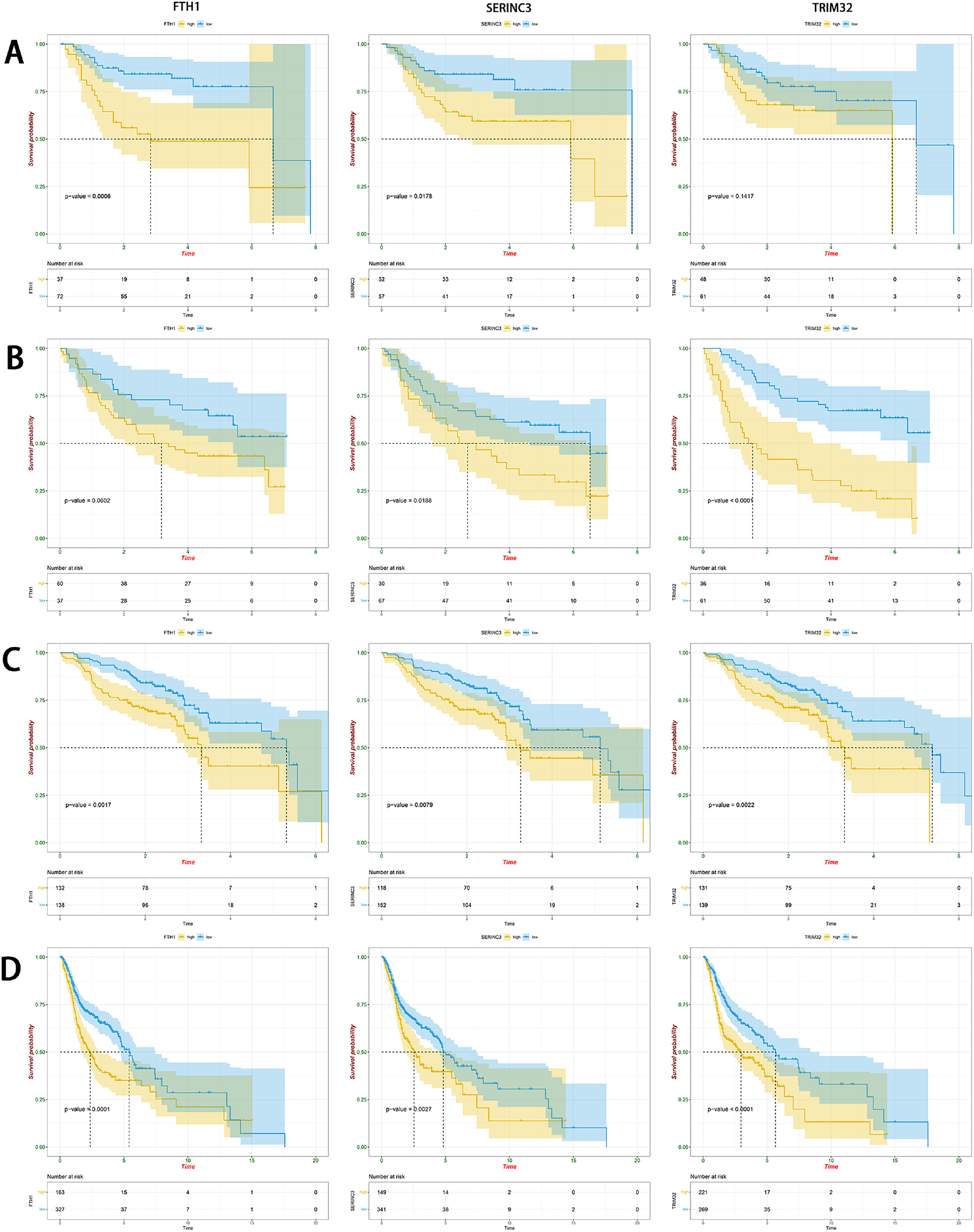

Supplement: Supplementary file 14 — Supplementary Fig. 15. The GSEA analysis of FCGR2A based on the MSigDB C5 collection and Genomes pan-cancer analysis. [file mmc14.jpg]

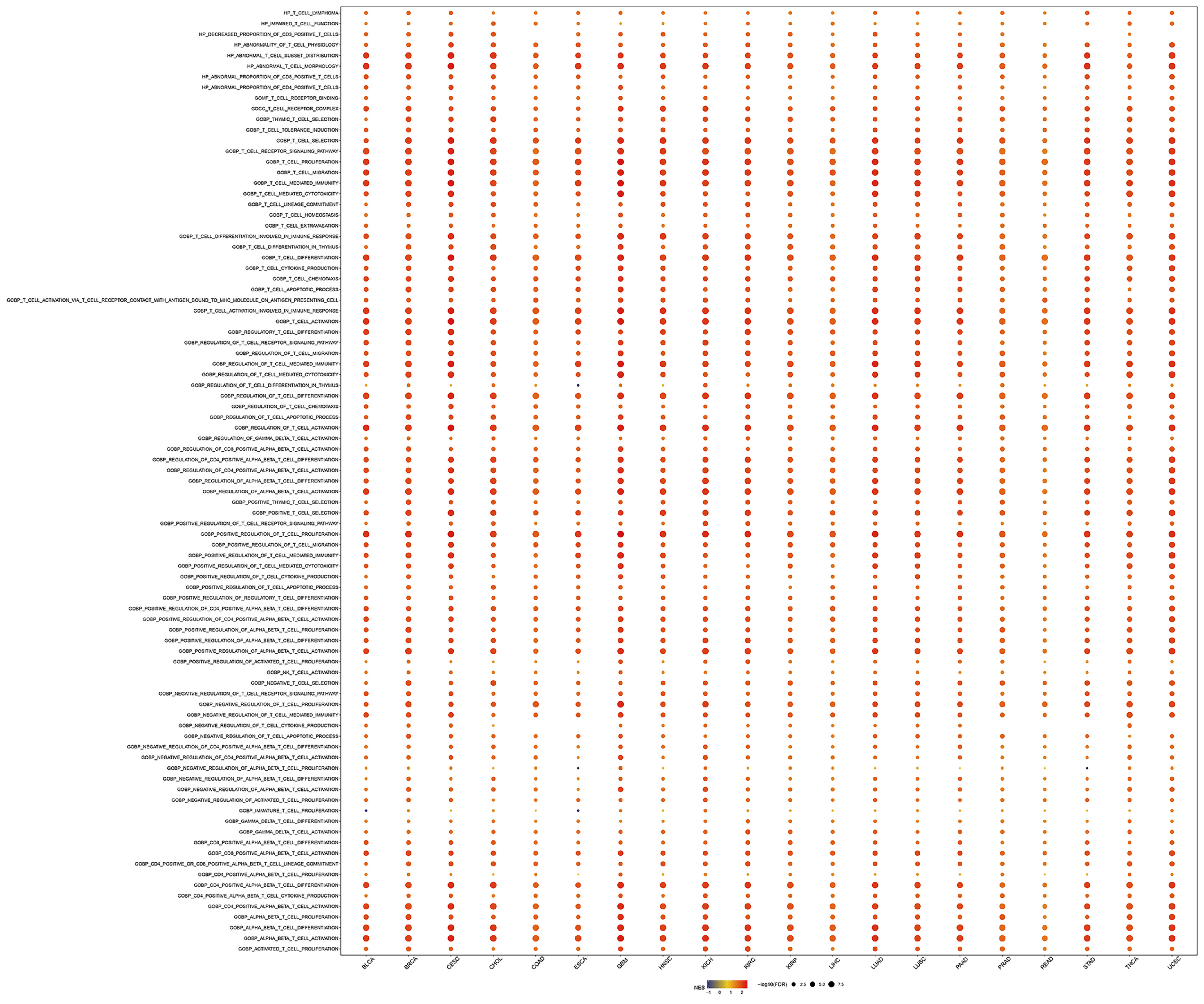

Supplement: Supplementary file 15 — Supplementary Fig. 16. Exploration of possibility treatment in molecular docking method. (A) Molecular docking of FCGR2A and SM-101. (B) Molecular docking of FCGR2A and Cytosine arabinoside. (C) Molecular docking of FCGR2A and Acetaminophen. (D) Molecular docking of FCGR2A and Cetuximab. [file mmc15.jpg]

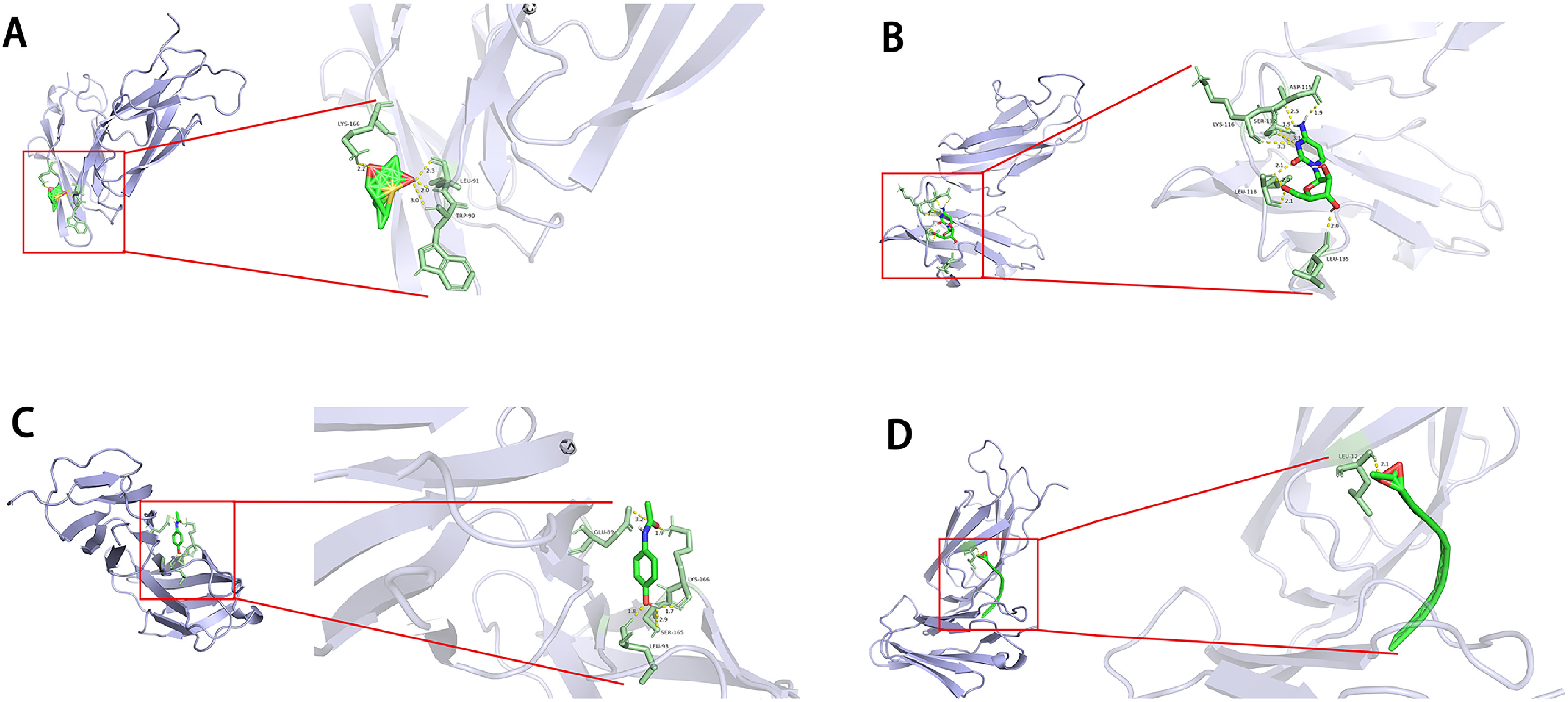

Supplement: Supplementary file 16 — Supplementary Fig. 17. The relationship between FCGR2A and clinical features. [file mmc16.jpg]

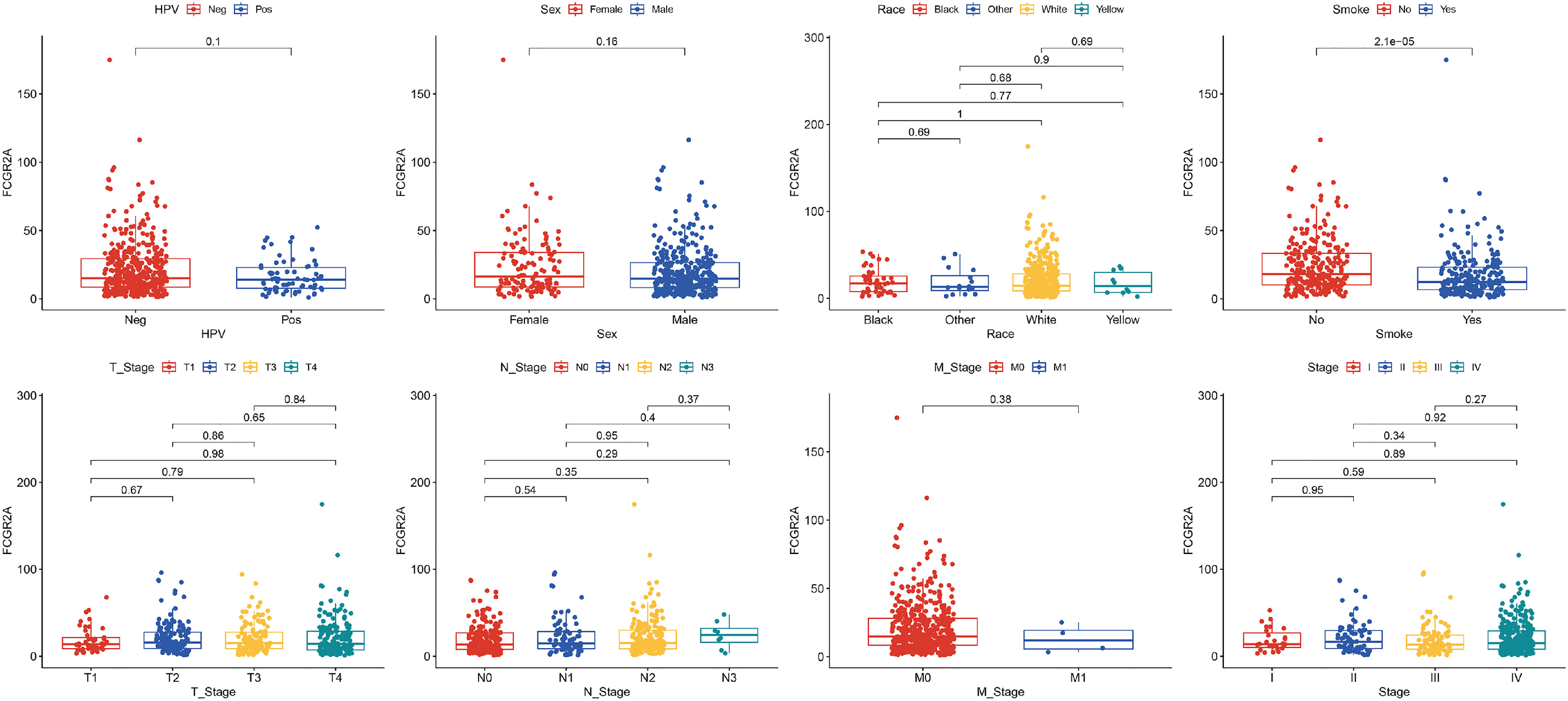

Supplement: Supplementary file 17 — Supplementary Fig. 18. The expression of FCGR2A in all the primary sites of HNSCC. [file mmc17.jpg]

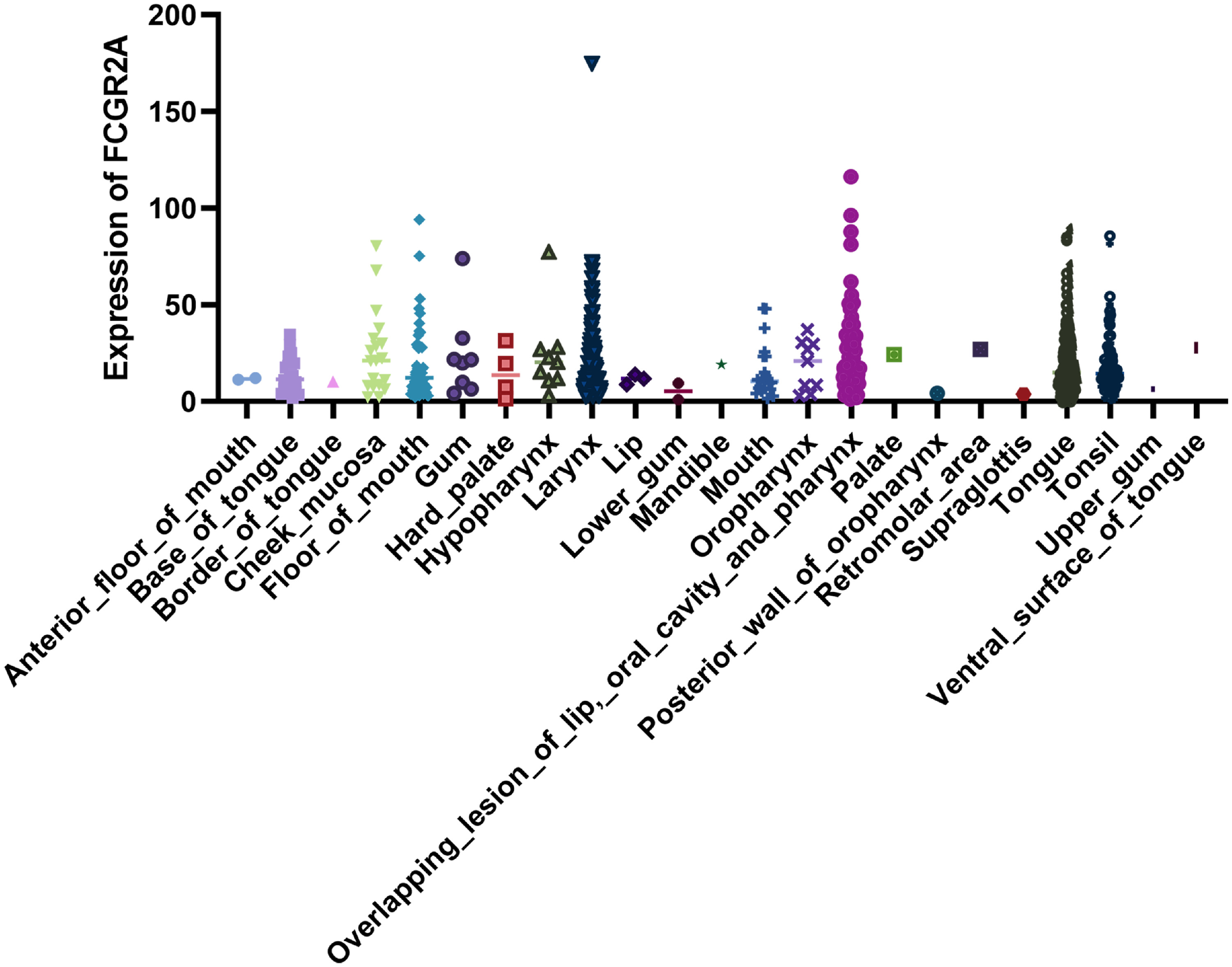

Supplement: Supplementary file 18 — Supplementary Table 1. The sequences of primers and siRNAs used in the research. [file mmc18.jpg]
