# Supplementary material for: Multi-omics comprehensive analyses of programmed cell death patterns to regulate the immune characteristics of head and neck squamous cell carcinoma
Source: Transl Oncol. 2024 Jan 18;41:101862. doi: 10.1016/j.tranon.2023.101862 (PMC10825548; doi:10.1016/j.tranon.2023.101862)
Supplement: Supplementary file 19 — Supplementary Table 2. The list of all genes from 14 PCD types. [file mmc19.docx]

| **Sequences of Primers** | |
| --- | --- |
| **FCGR2A** | **Forward:** TTTGAGATGAGTAATCCCAGCCA |
|  | **Reverse:** TCAGGCCCAGTCTCCATTTTA |
| **GAPDH** | **Forward:** GTCAGCCGCATCTT CTTT |
|  | **Reverse:** CGCCCAATACGACCAAAT |
| **Sequences of siRNAs** | |
| **si- FCGR2A #1** | **Forward:** GAGACCCAAAUGUCUCAGATT |
|  | **Reverse:** UCUGAGACAUUUGGGUCUCTT |
| **si- FCGR2A #2** | **Forward:** CAGGUUACAUGAGAGCAAUTT |
|  | **Reverse:** AUUGCUCUCAUGUAACCUGTT |
| **si- FCGR2A #3** | **Forward:** GGUCAUUGCGACUGCUGUATT |
|  | **Reverse:** UACAGCAGUCGCAAUGACCTT |
| **NC** | **Forward:** UUCUCCGAACGUGUCACGUTT |
|  | **Reverse:** ACGUGACACGUUCGGAGAATT |
